# Multiplexed CRISPR-mediated engineering of protein secretory pathway genes in the thermotolerant methylotrophic yeast *Ogataea thermomethanolica*

**DOI:** 10.1371/journal.pone.0261754

**Published:** 2021-12-23

**Authors:** Worarat Kruasuwan, Aekkachai Puseenam, Sutipa Tanapongpipat, Niran Roongsawang

**Affiliations:** Microbial Cell Factory Research Team, Biorefinery and Bioproduct Technology Research Group, National Center for Genetic Engineering and Biotechnology, National Science and Technology Development Agency, Khlong Nueng, Khlong Luang, Pathum Thani, Thailand; West China Hospital, Sichuan University, CHINA

## Abstract

CRISPR multiplex gRNA systems have been employed in genome engineering in various industrially relevant yeast species. The thermotolerant methylotrophic yeast *Ogataea thermomethanolica* TBRC 656 is an alternative host for heterologous protein production. However, the limited secretory capability of this yeast is a bottleneck for protein production. Here, we refined CRISPR-based genome engineering tools for simultaneous mutagenesis and activation of multiple protein secretory pathway genes to improve heterologous protein secretion. We demonstrated that multiplexed CRISPR-Cas9 mutation of up to four genes (*SOD1*, *VPS1*, *YPT7* and *YPT35*) in one single cell is practicable. We also developed a multiplexed CRISPR-dCas9 system which allows simultaneous activation of multiple genes in this yeast. 27 multiplexed gRNA combinations were tested for activation of three genes (*SOD1*, *VPS1* and *YPT7*), three of which were demonstrated to increase the secretion of fungal xylanase and phytase up to 29% and 41%, respectively. Altogether, our study provided a toolkit for mutagenesis and activation of multiple genes in *O*. *thermomethanolica*, which could be useful for future strain engineering to improve heterologous protein production in this yeast.

## Introduction

Clustered regularly interspaced short palindromic repeat (CRISPR) systems, originally discovered as an immunological mechanism in bacteria and archaea [1], are now widely used as genome editing tools. While many types of CRISPR systems have been discovered, CRISPR-associated protein 9 (Cas9), which is an RNA-guided endonuclease that cleaves target DNA, is the most commonly used for genome editing [2]. The genome editing process requires complementarity between the target site in the genome with Cas9-associated guide RNA (gRNA), which guides Cas9 to perform a double-strand break adjacent to a protospacer adjacent motif. Besides editing, an endonuclease-inactive Cas9 mutant (dCas9) can be employed to modulate gene expression in a gRNA-dependent manner. Fusion of dCas9 protein to either transcriptional repressors or activators enables efficient transcriptional regulation and is widely employed for gene interference (CRISPRi) and gene activation (CRISPRa) in several organisms [3].

CRISPR multiplex gRNA systems have been employed in genome engineering to mutate or modulate the expression of several target genes in one single cell, and this strategy is effective for genome engineering in a variety of yeast strains [3]. In *Saccharomyces cerevisiae*, the efficiency of editing two genes is about 65–87%, although efficiency declines for multiplex editing of three (57–75%) and four (15–27%) genes [4]. A similar efficiency was demonstrated for Cas9-mediated multiplex editing in the methylotrophic yeast *Pichia pastoris* [5], whereas markedly lower efficiency was reported for multiplex editing in another methylotrophic yeast *Ogataea parapolymorpha* and *O*. *polymorpha* [6,7]. In addition to editing, the expression of multiple yeast genes can be modulated in parallel with dCas9. In *Yarrowia lipolytica*, multiplexed CRISPRa with five gRNAs targeting the promoters of two native *β*-glucosidase genes led to increased proliferation and cellobiose consumption [8]. For enhanced production of bioproducts in yeasts, multiplexed CRISPRa has been combined with other systems including CRISPRi and gene deletion. For instance, control of *S*. *cerevisiae* gene expression in glycolysis and 2,3-butanediol pathways by repression (CRISPRi) of four genes and activation (CRISPRa) of the *BDH* gene in the one single cell resulted in increased 2,3-butanediol production [9]. Additionally, incorporation of CRISPRa, CRISPRi, and gene deletion for metabolic engineering in *S*. *cerevisiae* increased *β*-carotene production and display of endoglucanase on the yeast cell surface [10]. These studies highlight the power of multiplex CRISPR-based genome engineering approaches, which could be adopted in other industrially important, but less studied yeast species including *O*. *thermomethanolica*.

*O*. *thermomethanolica* TBRC 656 is thermotolerant methylotrophic yeast that is recognized as an alternative host for heterologous protein production [11–13]. Advantages of this host include strong methanol and sucrose-inducible promoters, ease of growth to high cell density using low-cost substrates like sucrose and molasses, and thermotolerance up to 40°C [14–17]. A drawback of *O*. *thermomethanolica* is its limited capability for protein secretion similar to other yeasts like *Kluyveromyces lactis* and *S*. *cerevisiae* [18–20]. To overcome secretory bottlenecks in heterologous protein production, fusion of native signal peptides for driving protein secretion and systems for overexpression of native chaperones have been employed in this strain [21]. CRISPR-based genome engineering has been established and demonstrated to be efficient in *O*. *thermomethanolica* for both gene mutagenesis [22] and activation [23]. In the latter study, gRNAs targeting promoter regions upstream of oxidative stress (*SOD1*) and vacuolar and protein sorting (*VPS1* and *YPT7*) genes were tested for mediating CRISPRa, which led to moderately enhanced secretion of fungal xylanase and phytase. All gRNAs were tested individually, and multiplex gRNAs were not explored.

In this study, we developed CRISPR multiplex gRNA systems for genome engineering in *O*. *thermomethanolica* towards the goal of improving heterologous protein secretion. Four genes involved in protein secretory pathways (*SOD1*, *VPS1*, *YPT7*, and *YPT35*) were targeted for either multiplex gene mutagenesis or activation by monitoring the secretion of model heterologous proteins, i.e., non-glycosylated fungal xylanase and glycosylated fungal phytase. Simultaneous mutagenesis or activation of multiple genes was demonstrated suggesting that the system could be a useful tool for the development of industrial *O*. *thermomethanolica* yeast cell factories for heterologous protein production.

## Materials and methods

### Strains, plasmids and culture conditions

The strains and plasmids used in this study are listed in Table 1. *Escherichia coli* strain DH5α was used for general cloning and grown at 37°C with agitation (250 rpm) in Luria-Bertani (LB) broth or LB agar plates with appropriate antibiotics. Strains Ot-Cas9-Xyl, Ot-dCas9-VP64-Xyl and Ot-dCas9-VP64-Phy were used as hosts for yeast transformation. All engineered yeast strains were cultivated on YPD medium (10 g L^–1^ yeast extract, 20 g L^–1^ peptone, and 20 g L^–1^ glucose) supplemented with appropriate antibiotics at 30°C with agitation (250 rpm).

**Table 1:** Strains and plasmids used in this study.

Strains and plasmids	Relevant characteristics	Source
**Strains**		
*E*. *coli* DH5α	Commercial host for cloning	
Ot-Cas9-Xyl	*O*. *thermomethanolica* harboring pOtAOX-Cas9 and fungal xylanase gene under the control of pMal promoter, G418^R^	[23]
Ot-dCas9-VP64-Xyl	*O*. *thermomethanolica* harboring pOtAOX-dCas9-VP64 and fungal xylanase gene under the control of pMal promoter, G418^R^	[23]
Ot-dCas9-VP64-Phy	*O*. *thermomethanolica* harboring pOtAOX-dCas9-VP64 and fungal phytase gene under the control of pMal promoter, G418^R^	[23]
**Plasmids**		
pOtAOX-Hyg	pOtAOX inducible expression vector, Hyg^R^	[22]
pOtAOX-gRNA	Inducible expression vector containing multiplexed gRNA cassettes (HH–20 bp specific determinant sequences–structural gRNA–HDV) under control of pOtAOX promoter, Hyg^R^	This study

### Construction of engineered yeasts expressing multiplexed gRNAs

To construct engineered yeasts for simultaneous gene mutagenesis or activation, Ot-Cas9-Xyl, Ot-dCas9-VP64-Xyl or Ot-dCas9-VP64-Phy strains (Table 1) were used as hosts for yeast transformation. gRNA cassettes for simultaneous gene mutagenesis were designed by assembling two, three or four gRNAs targeting *VPS1* (gRNA_*VPS1*_), *SOD1* (gRNA_*SOD1*_), *YPT35* (gRNA_*YPT35*_) and *YPT7* (gRNA_*YPT7*_) genes, respectively (S1 Table). For simultaneous gene activation, 27 gRNA cassettes (S2 Table) were created by combining gRNAs targeting either *VPS1* (gRNA1_*VPS1*_, gRNA2_*VPS1*_, gRNA3_*VPS1*_), *SOD1* (gRNA1_*SOD1*_, gRNA2_*SOD1*_, gRNA3_*SOD1*_), and *YPT7* (gRNA1_*YPT7*_, gRNA2_*YPT7*_, gRNA5_*YPT7*_) genes described in a previous report [23]. The gRNA cassettes used in this work were constructed as previously described [24] and synthesized by GenScript Biotech (Singapore) PTE. LTD. The pOtAOX-gRNA expression plasmids were constructed by insertion of the gRNA cassette fragment into pOtAOX-Hyg (Table 1) via the EcoRI and KpnI restriction sites. Constructed plasmids were verified by PCR using OtAOX-F and OtAOX-R primers and DNA sequencing (1st BASE, Singapore) using the OtAOX-R primer (S3 Table). To obtain engineered yeast expressing multiplexed gRNAs, BglII-linearized pOtAOX-gRNAs were either introduced into Ot-Cas9-Xyl, Ot-dCas9-VP64-Xyl or Ot-dCas9-VP64-Phy cells by electroporation with settings: electric field strength, 5.0 kV/cm; capacitance, 25 μF and resistance 400 Ω as described previously [13]. Transformants were then spread on YPD agar plates containing 100 μg mL^–1^ hygromycin. Plates were then incubated at 30°C for 2–3 days until colonies were observed. After that, engineered yeast strains were selected by PCR-amplification of the hygromycin resistance gene using Hyg-F and Hyg-R primers (S3 Table). Positive transformants were then selected for quantitative enzyme analysis.

### Verification of simultaneous gene mutagenesis

Five colonies of simultaneous gene disrupted mutants were selected for mutation analysis for each target gene. Yeast genomic DNA was extracted using a Wizard^®^ Genomic DNA Purification Kit (Promega Corporation, Madison, WI, USA). Target genes were PCR-amplified using gene-specific primers as indicated in S3 Table under the following amplification conditions: 98°C for 30 sec; 29 cycles of 98°C for 10 sec, 56.3°C for 30 sec, 72°C for 1 min and final extension at 72°C for 5 min. Sanger DNA sequencing was performed using gene-specific primers by 1st BASE DNA sequencing service (Singapore). The mutation efficiency was defined as the ratio of the desired mutants to the total tested colonies and calculated using the following formula: Percentage of mutation efficiency = (Number of desired mutants/Number of total tested colonies) ×100.

### Quantification of xylanase and phytase enzyme activity

Heterologous xylanase and phytase were expressed by growing the transformants in 5 mL YPD supplemented with 100 μg mL^–1^ zeocin at 30°C with agitation at 250 rpm for 24 h. Subsequently, the inoculum was transferred to 5 mL of YPS (10 g L^–1^ yeast extract, 20 g L^–1^ peptone, and 20 g L^–1^ sucrose) at the initial OD_600_ at 0.2 and incubated at 30°C with agitation at 250 rpm for 24 h. Ot-Cas9-Xyl, Ot-dCas9-VP64-Xyl or Ot-dCas9-VP64-Phy without gRNA were used as controls. 1 mL samples of cell suspension were analyzed for cell density by measuring OD_600_ nm using a SmartSpec Plus Spectrophotometer (BioRAD, USA) and activities of heterologous enzymes. Xylanase activity was measured by the DNS method [25] using 1% beechwood xylan (Sigma-Aldrich, MO, USA) as a substrate following a previously described protocol [26]. Phytase activity was assayed by measuring the release of inorganic orthophosphate from phytate [27] following a protocol described previously [28]. One unit of enzyme activity was defined as the amount of enzyme that liberates 1 μ mole per min of either reducing sugar or inorganic phosphate. For comparison of enzymes produced from different cultures, the enzymatic activities of different strains were normalized to the cell density measured as OD_600_ nm (U/OD).

### Stability determination of simultaneous gene mutagenesis and activation

To investigate the effect of simultaneous gene mutagenesis on cell growth, all positive transformants were cultivated in YPD medium supplemented with appropriate antibiotics at 30°C with agitation (250 rpm) for 48 h. 1 mL cell cultures were collected at every 12 h interval to measure the optical density (OD) at 600 nm for observation of cell growth by compared to the control (Ot-Cas9-Xyl).

To evaluate the long-term stability of simultaneous gene activation, positive transformants were cultivated in a nonselective YPD medium for 40 generations (20 days). 200 μL of culture broth was transferred to 5 mL YPD medium every 12 h and this procedure was repeated forty times. Every 10 generations, cells were collected and cultivated in YPS medium to quantify the activity of either xylanase or phytase enzymes following previously described.

### Quantitative real-time PCR analysis

Total RNA was isolated from yeast cells using an RNeasy Mini Kit (Qiagen, Germany). DNase I-treated RNA was then used as a template for first-strand cDNA synthesis using a Revert Aid First Strand cDNA Synthesis Kit with oligo (dT)_18_ primer following the manufacturer’s recommended protocol (Thermo Fisher Scientific, MA, USA). Quantitative real-time PCR analysis was performed using the CFX96 Touch Real-Time PCR Detection System (Bio-Rad). Reactions were carried out in a total volume of 10 μL containing 200 ng of cDNA, 2 μL of 1 μM of gene-specific forward and reverse primers (S3 Table) and 5 μL of iQ™ SYBR^®^ Green Supermix (Bio-Rad, CA, USA). Each sample was analyzed in technical triplicates and a no-template control for each pair of primers was included. The amplification conditions were 95°C for 4 min; 40 cycles of 95°C for 15 sec and appropriate annealing/extension temperature for 30 sec. At the end of the amplification cycle, a melting analysis was conducted to verify the specificity of the reaction by heating the amplified products from 55°C to 95°C with continuous fluorescent reading at 0.5°C increments. Relative gene expression was described in terms of fold change compared with the strain harboring empty vector (No gRNA). Endogenous *O*. *thermomethanolica* actin (*ACT*) gene was used as an internal control and the gene expression level was calculated following the 2^-ΔΔCt^ method [29].

### Statistical analysis

Data were presented as mean ± S.D. Post-hoc corrected *p*-values less than 0.05, 0.01 or 0.001 were considered significant. Data were calculated from either three or five yeast mutants per treatment in three experimental replicates. Asterisks (*) represent statistically-significant change in expression. Statistical analysis was performed using one-way ANOVA using post hoc Duncan’s multiple range test (IBM Statistic SPSS, version 23).

## Results

### Multiplexed CRISPR-mediated gene mutagenesis in *O*. *thermomethanolica*

**Fig 1:**
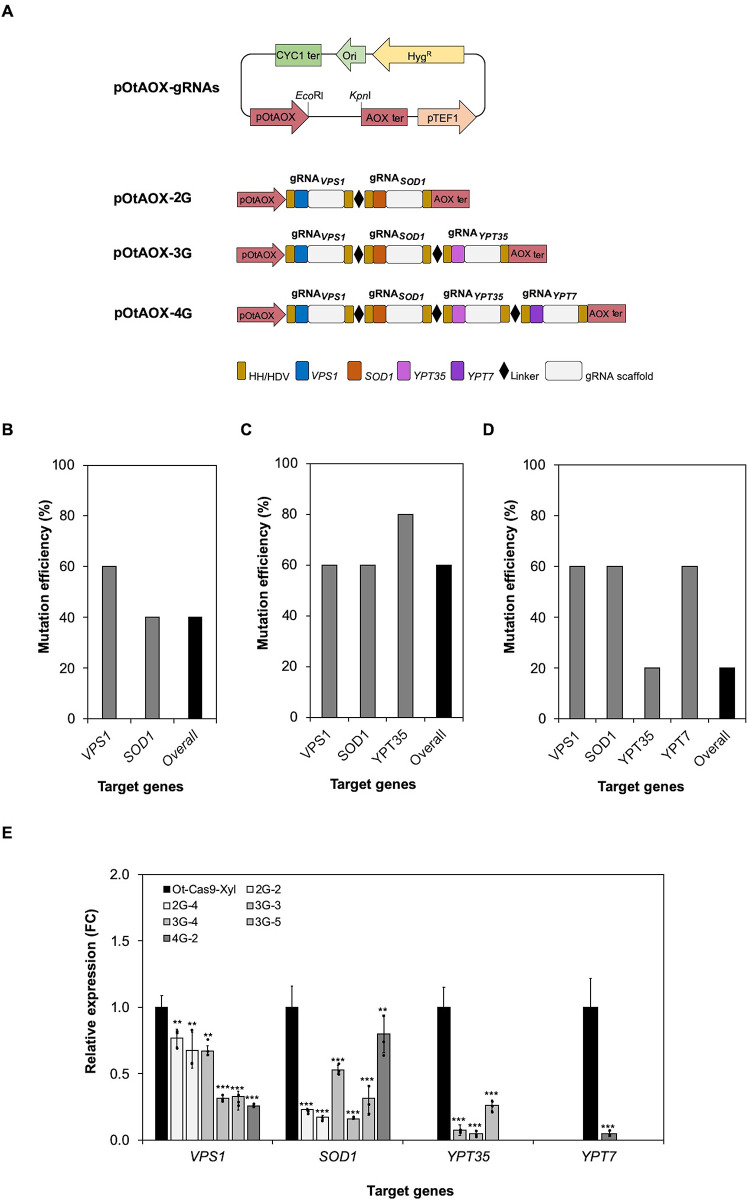
Multiplexed CRISPR-mediated gene mutagenesis in *O*. *thermomethanolica*. (A) Graphical representation of the pOtAOX-gRNA plasmid harbouring of either 2 gRNAs (2G; gRNA_*VPS1*_–gRNA_*SOD1*_), 3 gRNAs (3G; gRNA_*VPS1*_–gRNA_*SOD1*_–gRNA_*YPT35*_) or 4 gRNAs (4G; gRNA_*VPS1*_–gRNA_*SOD1*_–gRNA_*YPT35*_–gRNA_*YPT7*_) of protein secretory pathway genes driven by the Pol II promoter, pOtAOX. (B-D) Gene mutation efficiency of two, three and four protein secretory pathway genes in multiplex mutagenesis experiments calculated from five transformants. (E) Relative gene expression levels (fold change; FC) in simultaneous gene disrupted mutants. *O*. *thermomethanolica ACT* was used to normalize gene expression. Bars show mean ± S.D of three independent biological replicate experiments (*n* = 3); data from individual experiments are shown as black dots. Asterisks (*) indicate significantly altered expression profiles relative to Ot-Cas9-Xyl (**p<0.01 and ***p<0.001).

To explore the possibility of multiplex gene mutagenesis in *O*. *thermomethanolica*, 4 genes (*VPS1*, *SOD1*, *YPT7* and *YPT35*) involved in protein secretory pathway were simultaneously mutated and the impact of these mutations on secretion of non-glycosylated xylanase protein was examined (Fig 1). Three expression vectors for simultaneous gene mutation, i.e., 2G (gRNA_*VPS1*_–gRNA_*SOD1*_), 3G (gRNA_*VPS1*_–gRNA_*SOD1*_–gRNA_*YPT35*_) and 4G (gRNA_*VPS1*_–gRNA_*SOD1*_–gRNA_*YPT35*_–gRNA_*YPT7*_) (Fig 1A), were transformed into Ot-Cas9-Xyl and five transformant colonies were selected for target gene sequencing. Of three tested expression vectors, 3G exhibited the highest overall mutation efficiency (60%, 3/5 mutants), greater than either 2G (40%, 2/5 mutants) or 4G (20%, 1/5 mutants) vectors. Mutation of all individual genes was detected for each multiplex vector experiment, demonstrating that up to at least four genes can be mutated in one single cell (Fig 1B–1D, Table 2). Sequence analysis revealed that the introduction of multiplexed CRISPR-Cas9 into *O*. *thermomethanolica* resulted in either missense or nonsense mutations in target gene sequences (S1 Fig). The activity of heterologous xylanase was analyzed among transformants to demonstrate the effect of mutating these genes on protein secretion. In general, activity was lower among mutants compared with the control (Table 2). Transcriptional analysis of the target genes (*VPS1*, *SOD1*, *YPT7* and *YPT35*) in all positive simultaneous gene disrupted mutants were downregulated (Fig 1E, S4 Table). To investigate the effect of simultaneous gene mutagenesis on colony morphology and cell growth, all positive transformants were cultivated in YPD for 48 h. The 4G mutant showed a smaller colony size on the YPD agar plate than that of 2G, 3G and control (S2 Fig). The growth profile of 2G and 4G was marginally reduced at 12 and 24 h. However, the growth profile of the mutants did not differ from the control after 36 h except for 4G, which exhibited lower growth than other mutants and control (S3 Fig).

**Table 2:** Gene mutation efficiency and relative xylanase activity of the selected transformants.

Name (genes)	Clones	No. of mutated genes/total genes (mutated genes)	Gene mutation efficiency (%)	Relative activity (%U/OD)
Ot-Cas9-Xyl	Control			100 ± 2.84
2G (*vps1*–*sod1*)	1	1/2 (*vps1*)	50	68 ± 0.92
	2	2/2 (*vps1*–*sod1*)	100	77 ± 1.17
	3	0/2	0	90 ± 0.54
	4	2/2 (*vps1*–*sod1*)	100	87 ± 0.31
	5	0/2	0	96 ± 0.69
**Overall** (positive genotype)		**2/5**	**40**	**82 ± 6.97**
3G (*vps1*–*sod1*–*ypt35*)	1	1/3 (*ypt35*)	33.3	96 ± 1.27
	2	0/3	0	103 ± 1.14
	3	3/3 (*vps1*–*sod1*–*ypt35*)	100	93 ± 2.09
	4	3/3 (*vps1*–*sod1*–*ypt35*)	100	67 ± 0.86
	5	3/3 (*vps1*–*sod1*–*ypt35*)	100	80 ± 1.22
**Overall** (positive genotype)		**3/5**	**60**	**80 ± 13.05**
4G (*vps1*–*sod1*–*ypt35*–*ypt7*)	1	3/4 (*vps1–sod1–ypt7*)	75	57 ± 1.41
	2	4/4 (*vps1*–*sod1*–*ypt35*–*ypt7*)	100	48 ± 5.81
	3	0/4	0	93 ± 0.30
	4	2/4 (*sod1*–*ypt7*)	50	88 ± 0.26
	5	1/4 (*vps1*)	25	54 ± 0.92
**Overall** (positive genotype)		**1/5**	**20**	**48 ± 5.81**

Data are shown as number of mutated genes per total target genes and the percent gene mutation efficiency from five randomly selected colonies in each experiment (*n* = 5). Relative xylanase activity (%U/OD) are shown as mean ± S.D. from three independent biological replicate experiments (*n* = 3).

### Effect of multiple gene activation on xylanase expression

**Fig 2:**
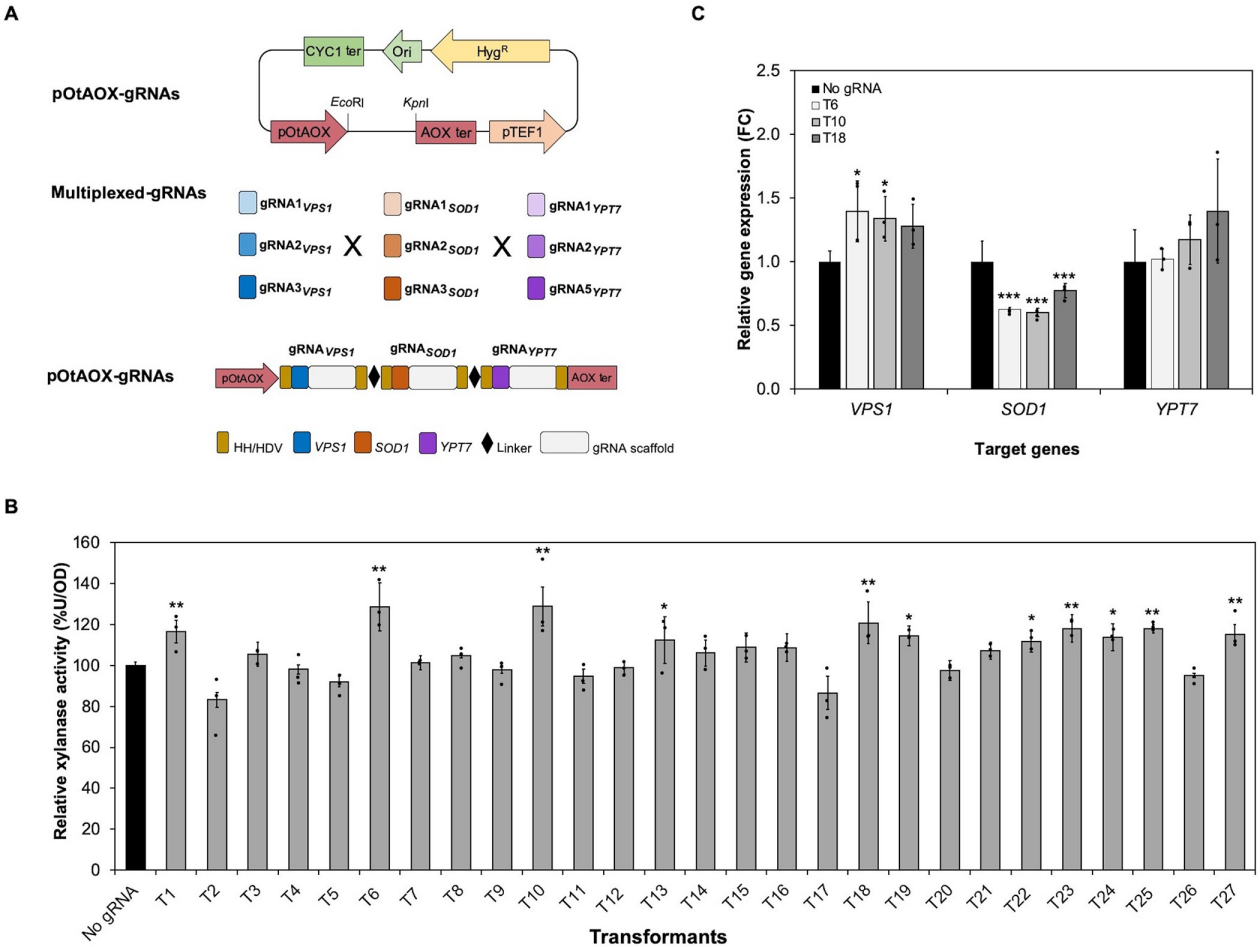
Effect of multiplex gene activation on xylanase expression in *O*. *thermomethanolica*. (A) Graphical representation of the pOtAOX-gRNA plasmid harbouring twenty-seven combinations (T1-T27) of three different gRNAs of *VPS1*, *SOD1* and *YPT7* genes driven by the Pol II promoter, pOtAOX. (B) Relative xylanase activity (%U/OD) of T1-T27 transformants. (C) Relative gene expression levels (fold change; FC) in T6, T10 and T18 transformants. *O*. *thermomethanolica ACT* was used to normalize gene expression. Bars show mean ± S.D of three independent biological replicate experiments (*n* = 3); data from individual experiments are shown as black dots. Asterisks (*) indicate significantly altered expression profiles relative to Ot-dCas9-VP64-Xyl without gRNA (No gRNA) (*p<0.05, **p<0.01 and ***p<0.001).

We demonstrated above that multiplexing two to four gRNAs results in the mutation of multiple target genes in one single cell. We therefore hypothesized that multiplexed gene activation (CRISPRa) was possible in yeast *O*. *thermomethanolica*. To test this hypothesis, 27 expression vectors for multiplex CRISPRa were designed by combining different gRNAs targeting *VPS1*, *SOD1* and *YPT7* genes (Fig 2A, S2 Table). Secretion of non-glycosylated heterologous xylanase was investigated in transformants carrying triple gene CRISPRa vectors. Cells transformed with 11 vectors (T1, T6, T10, T13, T18, T19, T22, T23, T24, T25 and T27) showed significantly increased secretion of xylanase compared with the control, with the greatest increase observed in T6 (29%), T10 (29%) and T18 (21%) (Fig 2B and S5 Table). Transcriptional analysis of the target genes in T6, T10 and T18 transformants showed corresponding activation patterns for *VPS1* and *YPT7* genes. The *SOD1* gene was significantly downregulated in all multiplex CRISPRa transformed cells (Fig 2C, S6 Table). In addition, stability of simultaneous gene activation on protein secretion in T6, T10 and T18 transformants was evaluated by culturing in a nonselective YPD medium for 40 generations. Our results showed that the relative xylanase activity was constant after long-term propagation in all multiplex CRISPRa transformants (S4A Fig).

### Effect of multiple gene activation on phytase expression

**Fig 3:**
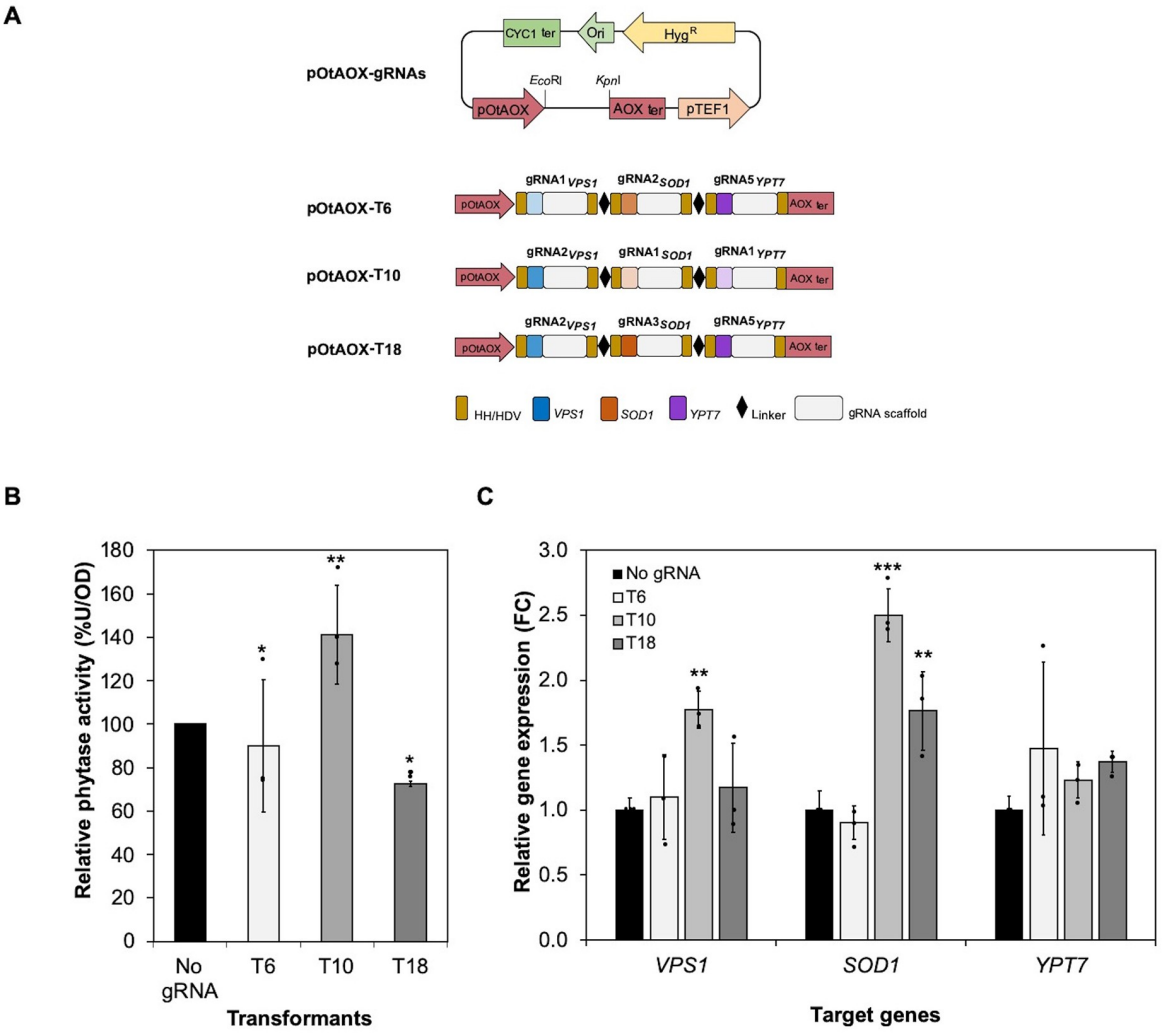
Effect of multiplex gene activation on phytase expression in *O*. *thermomethanolica*. (A) Graphical representation of the pOtAOX-gRNA plasmids harbouring three combinations (T6, T10 and T18) of three different gRNAs of *VPS1*, *SOD1* and *YPT7* genes driven by the Pol II promoter, pOtAOX. (B) Relative phytase activity (%U/OD) of T6, T10 and T18 transformants. (C) Relative gene expression levels (fold change; FC) in T6, T10 and T18 transformants. *O*. *thermomethanolica ACT* was used to normalize gene expression. Bars show mean ± S.D of three independent biological replicate experiments (*n* = 3); data from individual experiments are shown as black dots. Asterisks (*) indicate significantly altered expression profiles relative to Ot-dCas9-VP64-Phy without gRNA (No gRNA) (*p<0.05, **p<0.01 and ***p<0.001).

The three multiplex vectors associated with the highest secretion of xylanase (T6, T10, and T18) were introduced into the Ot-dCas9-VP64-Phy strain and the effect of multiplex CRISPRa on glycosylated phytase secretion was investigated. Of three multiplex constructs, significantly increased phytase secretion was observed only for T10 (41%) compared with the Ot-dCas9-VP64-Phy control (Fig 3B and S7 Table). *VPS1* gene expression was significantly upregulated in T10 cells. *SOD1* was also upregulated in T10 and T18 cells, whereas no significant change in expression was observed for *YPT7* in any transformed cell line (Fig 3C and S8 Table). Moreover, when growing the transformant in a nonselective YPD medium for 40 generations, we observed that the phytase secretion remained stable in T6, T10 and T18 transformants (S4B Fig), suggesting that multiplex CRISPRa system can be robustly used for multiplexed activation.

## Discussion

The thermotolerant methylotrophic yeast *O*. *thermomethanolica* TBRC 656 is an attractive host for heterologous protein production. In this work, we developed a CRISPR-based genome engineering system for multiplex mutation and activation of genes towards the goal of improving heterologous protein production in this yeast.

For testing simultaneous gene mutation, 4 protein secretory pathway genes (*SOD1*, *VPS1*, *YPT7* and *YPT35*) were selected as the model genes. Briefly, Vps1 and Ypt7 are proteins involved in protein transport from the late Golgi to the vacuole in the yeast cells [30]. Additionally, Ypt7 is also played important role in a major protein complex for recycling membrane proteins to the Golgi and plasma membrane in order to maintain the functionality of the vacuole [31]. Ypt35 is a PX domain-containing protein that is also reported in the protein transport process [32]. Whereas, Sod1, which is commonly known for its ROS scavenging activity, has also been documented as a helper protein for heterologous protein secretion in *K*. *lactis* [33]. Single mutagenesis of *SOD1*, *VPS1*, *YPT7*, and *YPT35* using CRISPR-Cas9 resulted in the reduction of xylanase secretion in *O*. *thermomethanolica* [23,32]. In this study, a mutation efficiency of 40% and 60% (two and three genes, respectively) was demonstrated for multiplex CRISPR-Cas9 mutation in *O*. *thermomethanolica*, which is markedly higher than that observed in the related methylotrophic yeasts *O*. *parapolymorpha* (2–5%) [6] and *O*. *polymorpha* (24%) [7]. However, the efficiency of mutating individual genes in multiplex gRNA experiments in *O*. *thermomethanolica* is lower than that observed when gRNAs are expressed individually [22,23,32]. Although there appears to be a trend of declining efficiency with increasing numbers of co-expressed gRNAs, more data are needed to determine the maximum number of gRNAs that can be co-expressed to generate mutations in all target genes in one single cell at a practically useful level. We found that multiplexed CRISPR-Cas9 mutagenesis frequently resulted in a frameshift and premature stop codon (nonsense mutation) for the *VPS1* gene. However, missense mutations were more common at the other target genes (*SOD1*, *YPT35*, and *YPT7*) (S1 Fig). Consistent with the xylanase activity, downregulation of all target genes was observed in all positive transformants (Fig 1E). Moreover, simultaneous gene mutagenesis was found to be closely linked to cell growth which was found in 4G (S2 and S3 Figs). However, it was observed that triple mutants (3G) obviously showed higher cell growth than double mutant (2G) at 12 h and 24 h (S3 Fig). This result suggests that suppressor mutation may be arisen in multiple mutations of *O*. *thermomethanolica*. Similarly, introduction of a *pmr1*, encoding a member of the P-type ATPase family, null mutation into the *sod1*Δ *sod2*Δ double mutant exhibited higher cell growth than those of double mutant in *S*. *cerevisiae* [34].

Since multiplexing gRNAs was shown to work for mutation of three genes in one single cell, we then tested whether multiplexing gRNAs is feasible for CRIPSRa in *O*. *thermomethanolica*. We tested gRNAs shown to mediate CRISPRa for *SOD1*, *VPS1*, and *YPT7* genes, in which up to 48% increases in target gene expression can be obtained for gRNAs expressed individually [23]. Since CRISPRa of the *SOD1*, *VPS1*, and *YPT7* genes individually results in modestly increased xylanase and phytase secretion [23], we hypothesized that multiplex CRISPRa of these genes could have an additive effect and lead to enhanced xylanase and phytase secretion compared with CRIPSRa of individual genes. However, we found that xylanase secretion was not markedly greater among any of the multiplex CRISPRa constructs (Fig 2B) compared with the earlier results of individual gene activation [23]. The effect of multiplex CRISPRa on phytase secretion was weaker, in which significantly enhanced secretion was observed for only the T10 multiplex CRISPRa construct (Fig 3B). These results suggest that the transcriptional activator employed in this study, VP64, is suboptimal for CRISPRa. Fusion of other activation domains such as VPR (VP64-p65-Rta) to dCas9 was reported to be more effective than VP64 in HEK 293T cells and *S*. *cerevisiae* [35,36]. The VPR activation domain is also functional for CRISPRa in the yeast *Y*. *lipolytica* [8], suggesting that alternative activation domains should be tested in *O*. *thermomethanolica*.

Having shown that cells transformed with some multiplex CRISPRa vectors demonstrated enhanced secretion of xylanase and phytase, corresponding increases in CRISPRa target gene expression were expected as shown previously for individual gene CRISPRa [23]. Consistent with expectation, upregulation of *VPS1* expression was observed for T6 and T10 multiplex CRISPRa vectors transformed into xylanase-expressing cells (Fig 2C) and the T10 multiplex CRISPRa vector transformed into phytase-expressing cells (Fig 3C). However, no change in *YPT7* expression was observed for any multiplex CRISPRa vector, and divergent results were obtained for *SOD1* expression. *SOD1* expression was downregulated in xylanase-expressing cells transformed with all three multiplex CRISPRa vectors tested (Fig 2C), but *SOD1* was upregulated (T10 and T18) or unchanged (T6) when the same vectors were transformed into phytase-expressing cells (Fig 3C). We do not know the reason for the divergent results for *SOD1*, but speculate that expression of heterologous xylanase exerts a different ER stress on the cell than phytase, which affects expression of ER stress-responsive genes [23,37].

## Conclusions

A CRISPR multiplex gRNA system was developed for genome engineering in *O*. *thermomethanolica*. We demonstrated that this system can be used for simultaneous mutation and activation of multiple protein secretory pathway genes in one single cell. Cells with CRISPR-induced mutations showed reduced secretion of heterologous non-glycosylated xylanase enzyme. In contrast, increased secretion of heterologous non-glycosylated xylanase and glycosylated phytase enzymes was observed in cells with CRISPR-activated genes. A limitation of the multiplex system is the reduced efficiency of mutation and activation compared with the individual gRNA system.

## Supporting information

S1 Fig Mutagenesis of protein secretory pathway genes of multiplexed gene mutants. (A) Multiplexed 2 gRNAs (2G, gRNA_*VPS1*_–gRNA_*SOD1*_). (B) Multiplexed 3 gRNAs (3G, gRNA_*VPS1*_–gRNA_*SOD1*_–gRNA_*YPT35*_). (C) Multiplexed 4 gRNAs (4G, gRNA_*VPS1*_–gRNA_*SOD1*_–gRNA_*YPT35*_–gRNA_*YPT7*_). The grey-highlighted letters indicate 20-bp specific determinant sequences of gRNA and protospacer adjacent motif (PAM) sequences are in bold letters. Asterisks (*) indicate premature stop codon, red letters indicate indel mutations and blue letters indicate amino acid substitution. Wild-type gene sequences are shown at the top of each alignment for comparison.
(DOCX)

S2 Fig Effect of simultaneous gene mutagenesis on morphology of yeast colony. All positive transformants were grown on YPD agar plate at 30°C for 48 h. Ot-Cas9-Xyl is control. 2G-2 and 2G-4 are 2G clone no.2 and no.4. 3G-3, 3G-4 and 3G-5 are 3G clone no. 3, no. 4 and no. 5, respectively. 4G-2 is 4G clone no. 2.
(DOCX)

S3 Fig Effect of simultaneous gene mutagenesis on cell growth. All positive transformants were grown on YPD at 30°C, 250 rpm for 48 h. Data are shown as mean ± S.D. from three independent biological replicate experiments (*n* = 3). Ot-Cas9-Xyl is control. 2G-2 and 2G-4 are 2G clone no.2 and no.4. 3G-3, 3G-4 and 3G-5 are 3G clone no. 3, no. 4 and no. 5, respectively. 4G-2 is 4G clone no. 2.
(DOCX)

S4 Fig Stability of simultaneous gene activation on xylanase (A) and phytase (B) secretion in T6, T10 and T18 transformants compared to either Ot-dCas9-VP64-Xyl or Ot-dCas9-VP64-Phy without gRNA (No gRNA). Relative activity (%U/OD) is shown as mean ± S.D. from three independent biological replicate experiments (*n* = 3).
(DOCX)

S1 Table List of gRNA cassettes used in this study for simultaneous gene mutagenesis by CRISPR-Cas9. Sequences of gRNA cassette fragment (HH–20 bp specific determinant sequences–structural gRNA–HDV) with the addition of EcoRI and KpnI restriction sites for pOtAOX-gRNA plasmids construction in this study. EcoRI (gaattc) and KpnI (ggtacc) sequences are in blue, the 20-bp specific determinant sequences of gRNA are in green, six nucleotides complementary to six nucleotides of targeted sequences are in red and the sequences of HH and HDV ribozymes, structural gRNA sequences and linker sequences are in dark blue, black and bold, respectively.
(DOCX)

S2 Table List of gRNA cassettes used in this study for multiplex gene activation by CRISPR-dCas9. Sequences of gRNA cassette fragment (HH–20 bp specific determinant sequences–structural gRNA–HDV) with the addition of EcoRI and KpnI restriction sites for pOtAOX-gRNA plasmids construction in this study. EcoRI (gaattc) and KpnI (ggtacc) sequences are in blue, the 20-bp specific determinant sequences of gRNA are in green, six nucleotides complementary to six nucleotides of targeted promoter sequences are in red and the sequences of HH and HDV ribozymes, structural gRNA sequences and linker sequences are in dark blue, black and bold, respectively.
(DOCX)

S3 Table List of primer sequences used in this study. (DOCX)

S4 Table Relative gene expression levels of simultaneous gene disrupted mutants. *O*. *thermomethanolica ACT* was used to normalize gene expression. Data are shown as mean ± S.D. from three independent biological replicate experiments (*n* = 3).
(DOCX)

S5 Table Xylanase activity of Ot-dCas9-VP64-Xyl expressing various gRNAs (T1-T27). Data are shown as mean ± S.D. from three independent biological replicate experiments (*n* = 3).
(DOCX)

S6 Table Relative gene expression levels of Ot-dCas9-VP64-Xyl expressing T6, T10 and T18 gRNAs. *O*. *thermomethanolica ACT* was used to normalize gene expression. Data are shown as mean ± S.D. from three independent biological replicate experiments (*n* = 3).
(DOCX)

S7 Table Phytase activity of Ot-dCas9-VP64-Phy expressing T6, T10 and T18 gRNAs. Data are shown as mean ± S.D. from three-independent biological replicate experiments (*n* = 3).
(DOCX)

S8 Table Relative gene expression levels of Ot-dCas9-VP64-Phy expressing T6, T10 and T18 gRNAs. *O*. *thermomethanolica ACT* was used to normalize gene expression. Data are shown as mean ± S.D. from three independent biological replicate experiments (*n* = 3).
(DOCX)
