# Supplementary material for: Multiplexed CRISPR-mediated engineering of protein secretory pathway genes in the thermotolerant methylotrophic yeast Ogataea thermomethanolica
Source: PLoS One. 2021 Dec 23;16(12):e0261754. doi: 10.1371/journal.pone.0261754 (PMC8699913; doi:10.1371/journal.pone.0261754)
Supplement: S1 Fig — (A) Multiplexed 2 gRNAs (2G, gRNAVPS1–gRNASOD1). (B) Multiplexed 3 gRNAs (3G, gRNAVPS1–gRNASOD1–gRNAYPT35). (C) Multiplexed 4 gRNAs (4G, gRNAVPS1–gRNASOD1–gRNAYPT35–gRNAYPT7). The grey-highlighted letters indicate 20-bp specific determinant sequences of gRNA and protospacer adjacent motif (PAM) sequences are in bold letters. Asterisks (*) indicate premature stop codon, red letters indicate indel mutations and blue letters indicate amino acid substitution. Wild-type gene sequences are shown at the top of each alignment for comparison. (DOCX) [file pone.0261754.s001.docx]

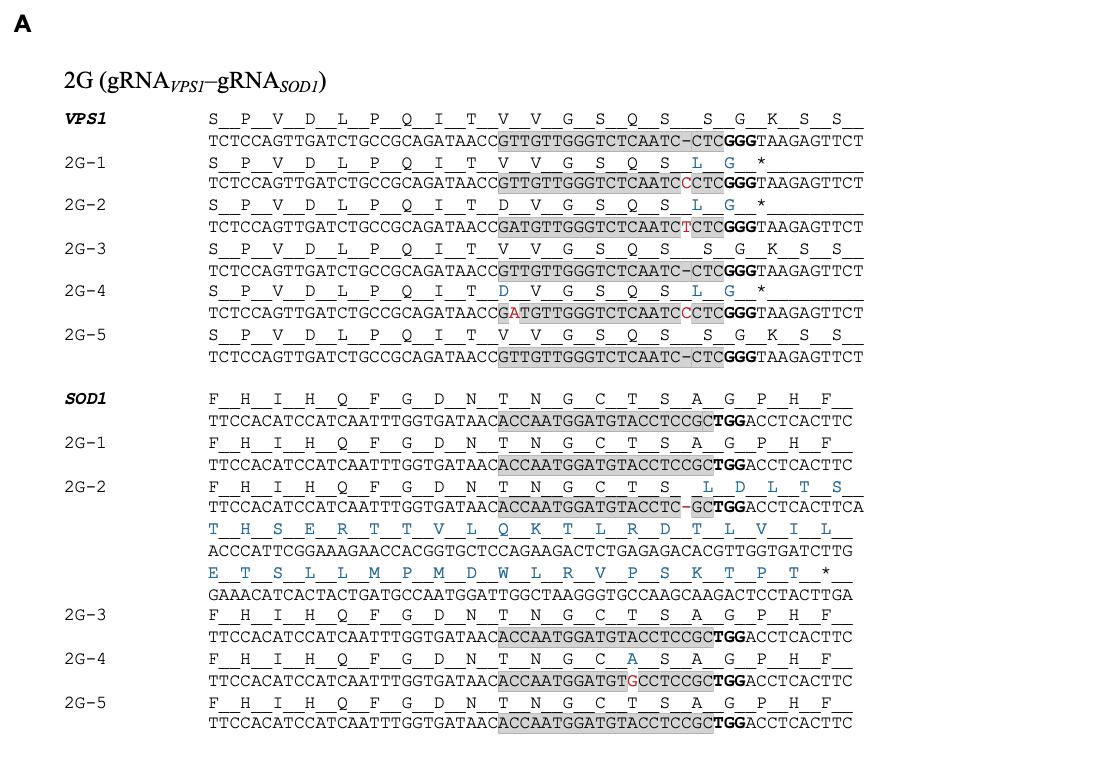


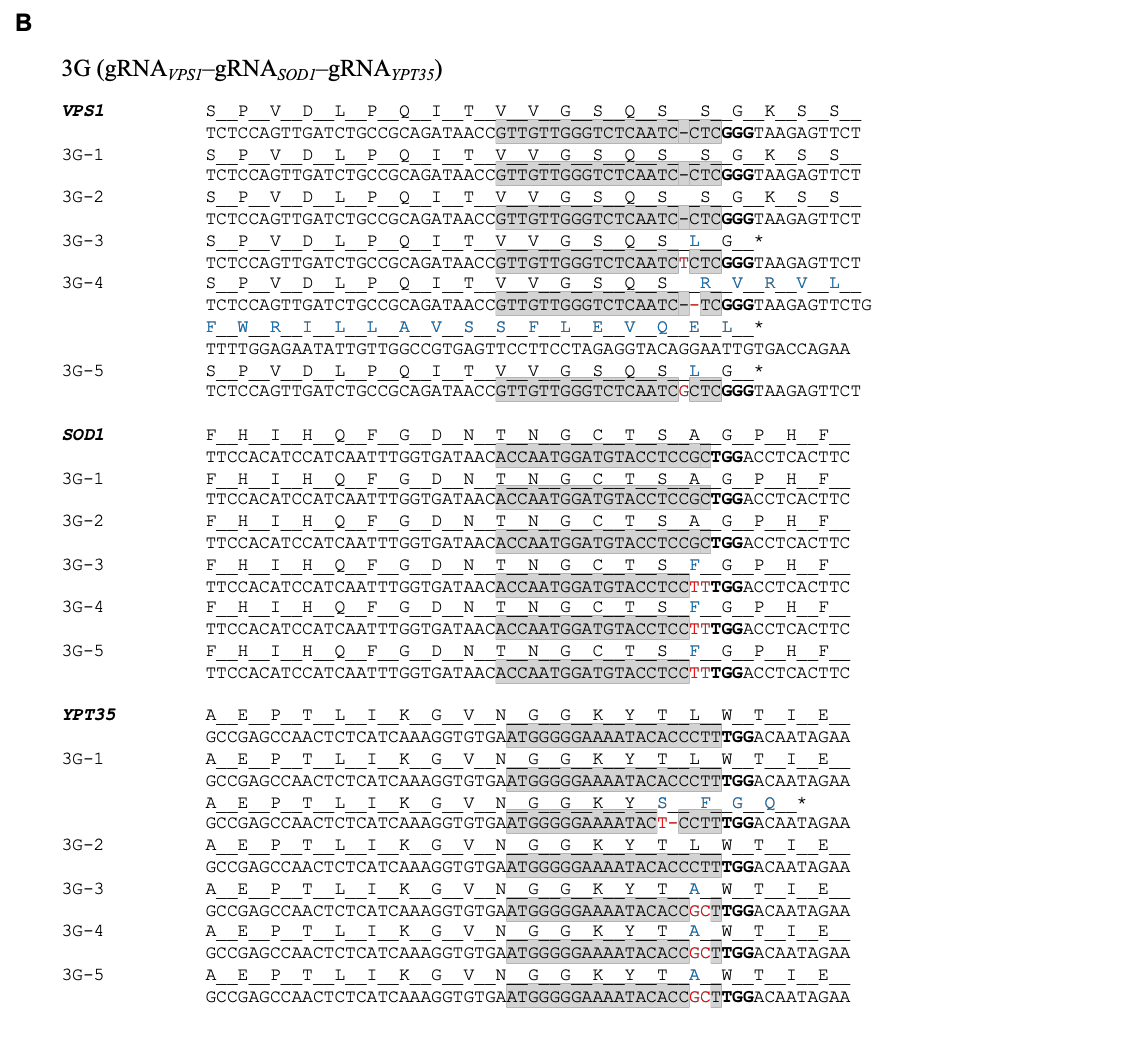


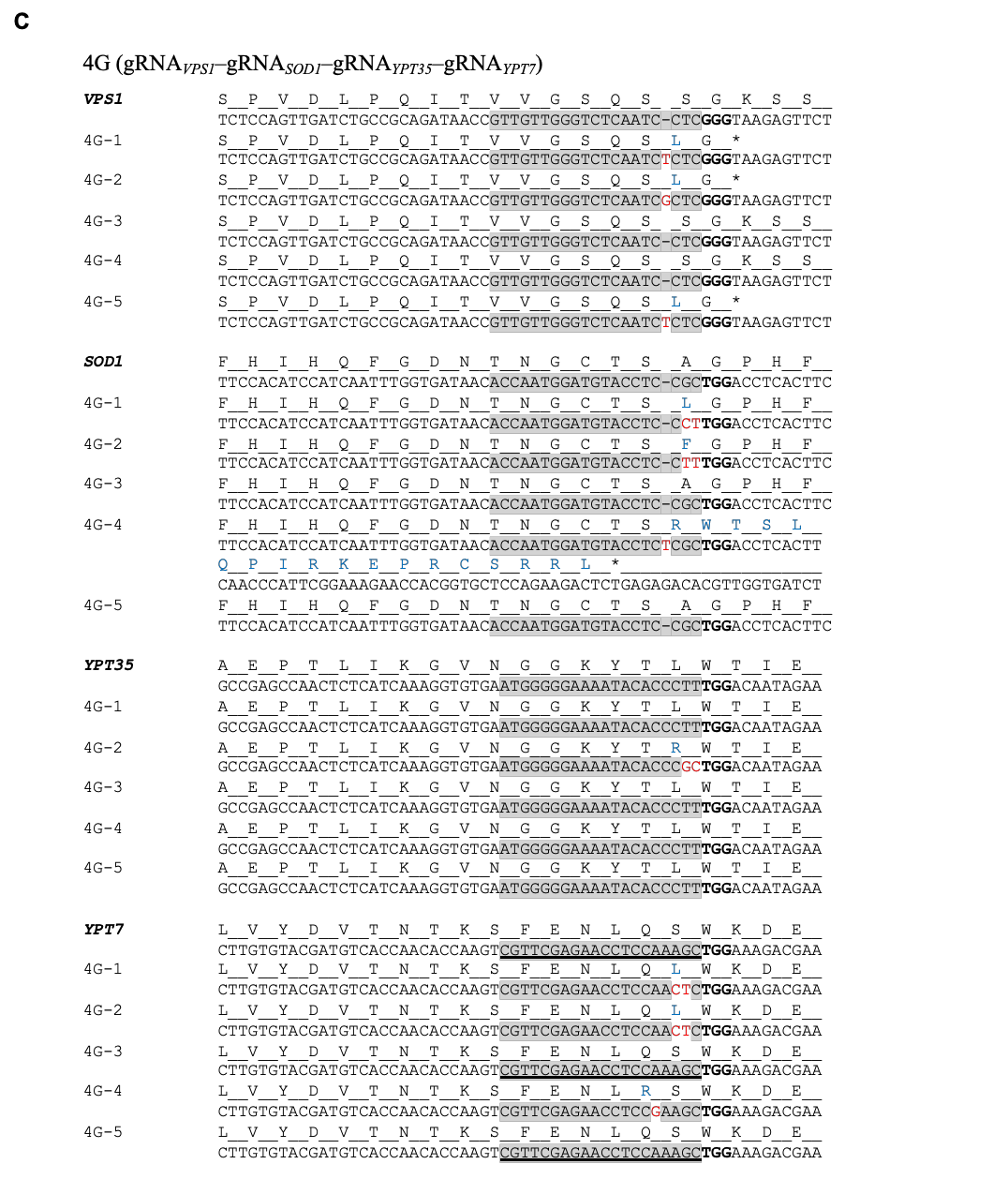


**Fig S1 Mutagenesis of protein secretory pathway genes of multiplexed gene mutants.**

(A) Multiplexed 2 gRNAs (2G, gRNA*_VPS1_*–gRNA*_SOD1_*). (B) Multiplexed 3 gRNAs (3G, gRNA*_VPS1_–*gRNA*_SOD1_*–gRNA*_YPT35_*). (C) Multiplexed 4 gRNAs (4G, gRNA*_VPS1_–*gRNA*_SOD1_*–gRNA*_YPT35_*–gRNA*_YPT7_*). The grey-highlighted letters indicate 20-bp specific determinant sequences of gRNA and protospacer adjacent motif (PAM) sequences are in bold letters. Asterisks (*) indicate premature stop codon, red letters indicate indel mutations and blue letters indicate amino acid substitution. Wild-type gene sequences are shown at the top of each alignment for comparison.
