# Supplementary material for: Multiplexed CRISPR-mediated engineering of protein secretory pathway genes in the thermotolerant methylotrophic yeast Ogataea thermomethanolica
Source: PLoS One. 2021 Dec 23;16(12):e0261754. doi: 10.1371/journal.pone.0261754 (PMC8699913; doi:10.1371/journal.pone.0261754)
Supplement: S2 Fig — All positive transformants were grown on YPD agar plate at 30°C for 48 h. Ot-Cas9-Xyl is control. 2G-2 and 2G-4 are 2G clone no.2 and no.4. 3G-3, 3G-4 and 3G-5 are 3G clone no. 3, no. 4 and no. 5, respectively. 4G-2 is 4G clone no. 2. (DOCX) [file pone.0261754.s002.docx]

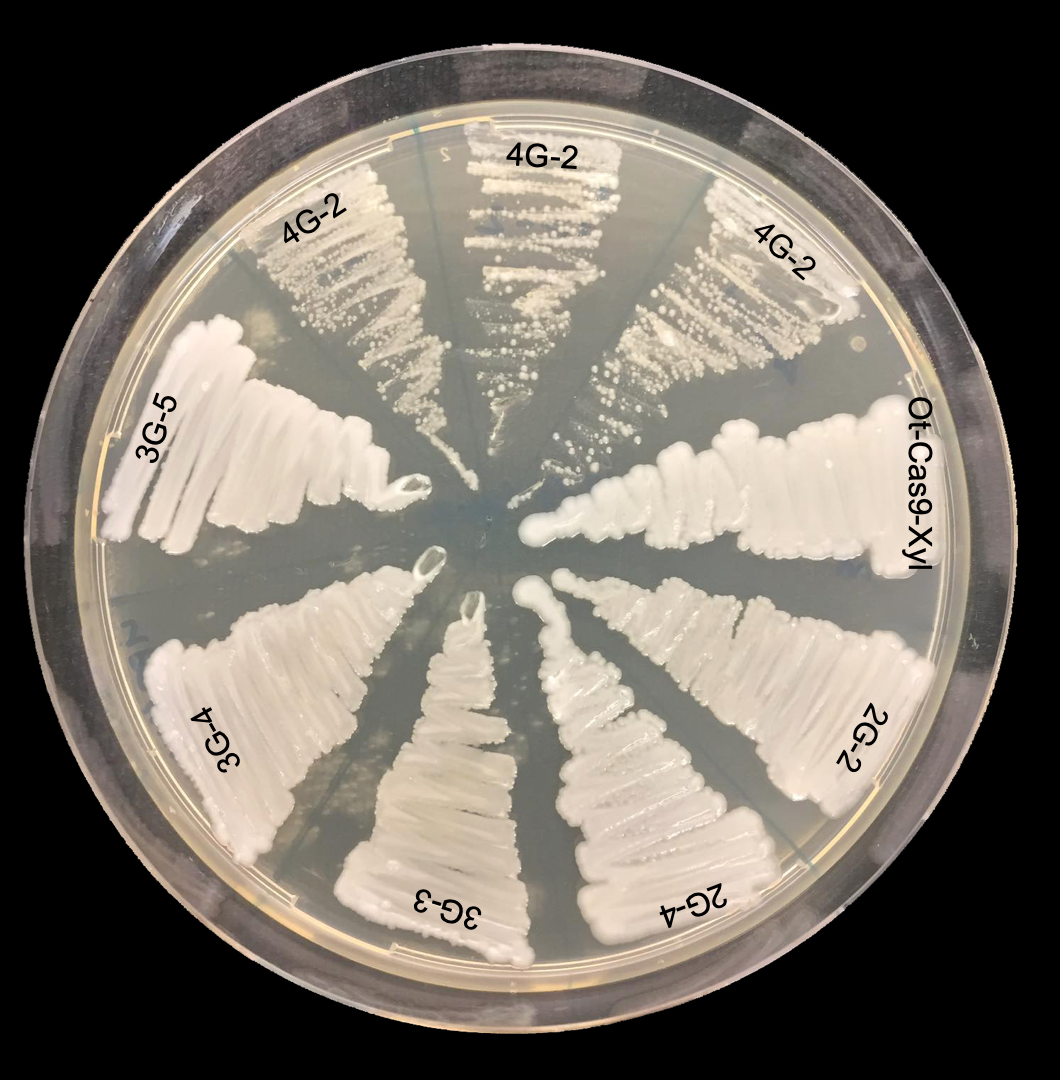


**Fig S2 Effect of simultaneous gene mutagenesis on morphology of yeast colony.** All positive transformants were grown on YPD agar plate at 30 ºC for 48 h. Ot-Cas9-Xyl is control. 2G-2 and 2G-4 are 2G clone no.2 and no.4. 3G-3, 3G-4 and 3G-5 are 3G clone no. 3, no. 4 and no. 5, respectively. 4G-2 is 4G clone no. 2.
