# Supplementary material for: Multiplexed CRISPR-mediated engineering of protein secretory pathway genes in the thermotolerant methylotrophic yeast Ogataea thermomethanolica
Source: PLoS One. 2021 Dec 23;16(12):e0261754. doi: 10.1371/journal.pone.0261754 (PMC8699913; doi:10.1371/journal.pone.0261754)
Supplement: S4 Fig — Stability of simultaneous gene activation on xylanase (A) and phytase (B) secretion in T6, T10 and T18 transformants compared to either Ot-dCas9-VP64-Xyl or Ot-dCas9-VP64-Phy without gRNA (No gRNA). Relative activity (%U/OD) is shown as mean ± S.D. from three independent biological replicate experiments (n = 3). (DOCX) [file pone.0261754.s004.docx]

**A**


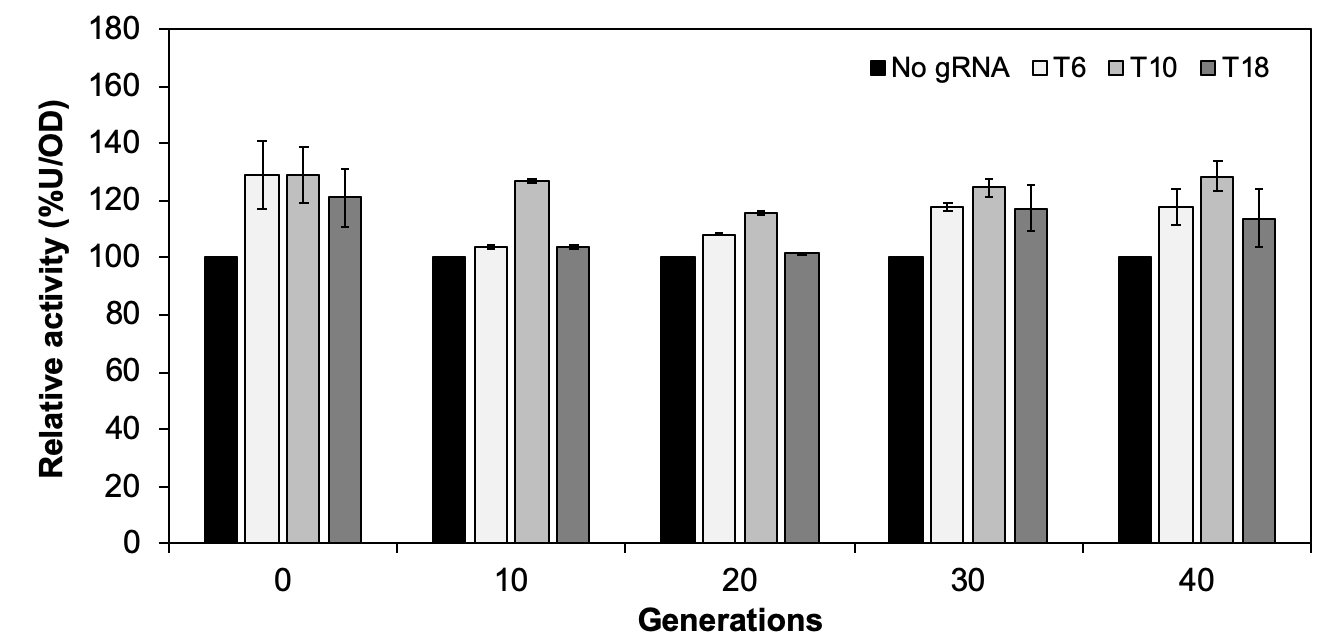


**B**


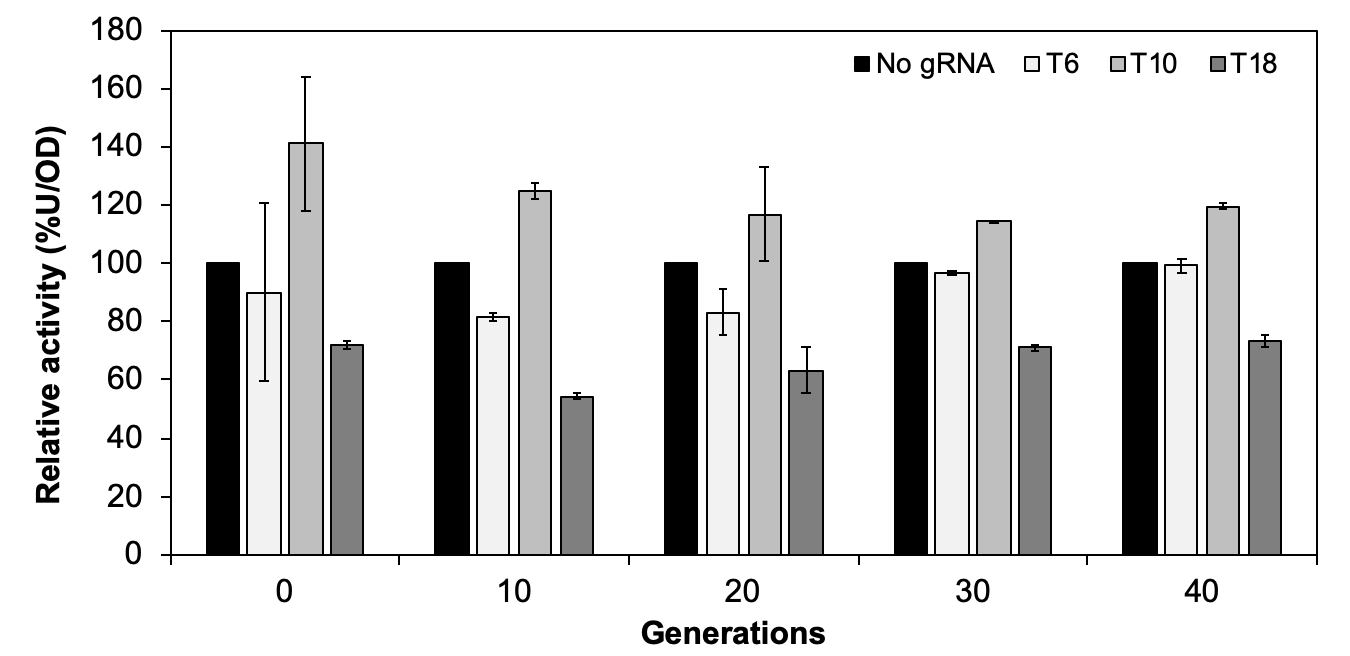


**Fig S4 Stability of simultaneous gene activation on xylanase (A) and phytase (B) secretion in T6, T10 and T18 transformants compared to either Ot-dCas9-VP64-Xyl or Ot-dCas9-VP64-Phy without gRNA (No gRNA).** Relative activity (%U/OD) is shown as mean ± S.D. from three independent biological replicate experiments (*n*=3).
