# Supplementary material for: Multiplexed CRISPR-mediated engineering of protein secretory pathway genes in the thermotolerant methylotrophic yeast Ogataea thermomethanolica
Source: PLoS One. 2021 Dec 23;16(12):e0261754. doi: 10.1371/journal.pone.0261754 (PMC8699913; doi:10.1371/journal.pone.0261754)
Supplement: S1 Table — Sequences of gRNA cassette fragment (HH–20 bp specific determinant sequences–structural gRNA–HDV) with the addition of EcoRI and KpnI restriction sites for pOtAOX-gRNA plasmids construction in this study. EcoRI (gaattc) and KpnI (ggtacc) sequences are in blue, the 20-bp specific determinant sequences of gRNA are in green, six nucleotides complementary to six nucleotides of targeted sequences are in red and the sequences of HH and HDV ribozymes, structural gRNA sequences and linker sequences are in dark blue, black and bold, respectively. (DOCX) [file pone.0261754.s005.docx]

**Table S1 List of gRNA cassettes used in this study for simultaneous gene mutagenesis by CRISPR-Cas9.** Sequences of gRNA cassette fragment (HH–20 bp specific determinant sequences–structural gRNA–HDV) with the addition of EcoRI and KpnI restriction sites for pOtAOX-gRNA plasmids construction in this study. EcoRI (gaattc) and KpnI (ggtacc) sequences are in blue, the 20-bp specific determinant sequences of gRNA are in green, six nucleotides complementary to six nucleotides of targeted sequences are in red and the sequences of HH and HDV ribozymes, structural gRNA sequences and linker sequences are in dark blue, black and bold, respectively.

| **Name** | **gRNAs** | **gRNA cassette sequences (5′ to 3′)** |
| --- | --- | --- |
| 2G | gRNA*_VPS1_*–gRNA*_SOD1_* | gaattcaacaacctgatgagtccgtgaggacgaaacgagtaagctcgtcgttgttgggtctcaatcctcgttttagagctagaaatagcaagttaaaataaggctagtccgttatcaacttgaaaaagtggcaccgagtcggtgcttttggccggcatggtcccagcctcctcgctggcgccggctgggcaacatgcttcggcatggcgaatgggac**aatcactagt**attggtctgatgagtccgtgaggacgaaacgagtaagctcgtcaccaatggatgtacctccgcgttttagagctagaaatagcaagttaaaataaggctagtccgttatcaacttgaaaaagtggcaccgagtcggtgcttttggccggcatggtcccagcctcctcgctggcgccggctgggcaacatgcttcggcatggcgaatgggacggtacc |
| 3G | gRNA*_VPS1_–* gRNA*_SOD1_*– gRNA*_YPT35_* | gaattcaacaacctgatgagtccgtgaggacgaaacgagtaagctcgtcgttgttgggtctcaatcctcgttttagagctagaaatagcaagttaaaataaggctagtccgttatcaacttgaaaaagtggcaccgagtcggtgcttttggccggcatggtcccagcctcctcgctggcgccggctgggcaacatgcttcggcatggcgaatgggac**aatcactagt**attggtctgatgagtccgtgaggacgaaacgagtaagctcgtcaccaatggatgtacctccgcgttttagagctagaaatagcaagttaaaataaggctagtccgttatcaacttgaaaaagtggcaccgagtcggtgcttttggccggcatggtcccagcctcctcgctggcgccggctgggcaacatgcttcggcatggcgaatgggac**aatcactagt**ccccatctgatgagtccgtgaggacgaaacgagtaagctcgtcatgggggaaaatacacccttgttttagagctagaaatagcaagttaaaataaggctagtccgttatcaacttgaaaaagtggcaccgagtcggtgcttttggccggcatggtcccagcctcctcgctggcgccggctgggcaacatgcttcggcatggcgaatgggacggtacc |
| 4G | gRNA*_VPS1_–* gRNA*_SOD1_*– gRNA*_YPT35_*– gRNA*_YPT7_* | gaattcaacaacctgatgagtccgtgaggacgaaacgagtaagctcgtcgttgttgggtctcaatcctcgttttagagctagaaatagcaagttaaaataaggctagtccgttatcaacttgaaaaagtggcaccgagtcggtgcttttggccggcatggtcccagcctcctcgctggcgccggctgggcaacatgcttcggcatggcgaatgggac**aatcactagt**attggtctgatgagtccgtgaggacgaaacgagtaagctcgtcaccaatggatgtacctccgcgttttagagctagaaatagcaagttaaaataaggctagtccgttatcaacttgaaaaagtggcaccgagtcggtgcttttggccggcatggtcccagcctcctcgctggcgccggctgggcaacatgcttcggcatggcgaatgggac**aatcactagt**ccccatctgatgagtccgtgaggacgaaacgagtaagctcgtcatgggggaaaatacacccttgttttagagctagaaatagcaagttaaaataaggctagtccgttatcaacttgaaaaagtggcaccgagtcggtgcttttggccggcatggtcccagcctcctcgctggcgccggctgggcaacatgcttcggcatggcgaatgggac**aatcactagt**cgaacgctgatgagtccgtgaggacgaaacgagtaagctcgtccgttcgagaacctccaaagcgttttagagctagaaatagcaagttaaaataaggctagtccgttatcaacttgaaaaagtggcaccgagtcggtgcttttggccggcatggtcccagcctcctcgctggcgccggctgggcaacatgcttcggcatggcgaatgggacggtacc |
