# Supplementary material for: Multiplexed CRISPR-mediated engineering of protein secretory pathway genes in the thermotolerant methylotrophic yeast Ogataea thermomethanolica
Source: PLoS One. 2021 Dec 23;16(12):e0261754. doi: 10.1371/journal.pone.0261754 (PMC8699913; doi:10.1371/journal.pone.0261754)
Supplement: S2 Table — Sequences of gRNA cassette fragment (HH–20 bp specific determinant sequences–structural gRNA–HDV) with the addition of EcoRI and KpnI restriction sites for pOtAOX-gRNA plasmids construction in this study. EcoRI (gaattc) and KpnI (ggtacc) sequences are in blue, the 20-bp specific determinant sequences of gRNA are in green, six nucleotides complementary to six nucleotides of targeted promoter sequences are in red and the sequences of HH and HDV ribozymes, structural gRNA sequences and linker sequences are in dark blue, black and bold, respectively. (DOCX) [file pone.0261754.s006.docx]

**Table S2 List of gRNA cassettes used in this study for multiplex gene activation by CRISPR-dCas9.** Sequences of gRNA cassette fragment (HH–20 bp specific determinant sequences–structural gRNA–HDV) with the addition of EcoRI and KpnI restriction sites for pOtAOX-gRNA plasmids construction in this study. EcoRI (gaattc) and KpnI (ggtacc) sequences are in blue, the 20-bp specific determinant sequences of gRNA are in green, six nucleotides complementary to six nucleotides of targeted promoter sequences are in red and the sequences of HH and HDV ribozymes, structural gRNA sequences and linker sequences are in dark blue, black and bold, respectively.

| **Name** | **gRNAs** | **gRNA cassette sequences (5′ to 3′)** |
| --- | --- | --- |
| T1 | gRNA1*_VPS1_*–gRNA1*_SOD1_*–gRNA1*_YPT7_* | gaattccccaacctgatgagtccgtgaggacgaaacgagtaagctcgtcgttgggaggcgcttggtgtcgttttagagctagaaatagcaagttaaaataaggctagtccgttatcaacttgaaaaagtggcaccgagtcggtgcttttggccggcatggtcccagcctcctcgctggcgccggctgggcaacatgcttcggcatggcgaatgggac**aatcactagt**aaggttctgatgagtccgtgaggacgaaacgagtaagctcgtcaaccttgatgagcaagtttggttttagagctagaaatagcaagttaaaataaggctagtccgttatcaacttgaaaaagtggcaccgagtcggtgcttttggccggcatggtcccagcctcctcgctggcgccggctgggcaacatgcttcggcatggcgaatgggac**aatcactagt**gacattctgatgagtccgtgaggacgaaacgagtaagctcgtcaatgtcaatgtggccattccgttttagagctagaaatagcaagttaaaataaggctagtccgttatcaacttgaaaaagtggcaccgagtcggtgcttttggccggcatggtcccagcctcctcgctggcgccggctgggcaacatgcttcggcatggcgaatgggacggtacc |
| T2 | gRNA1*_VPS1_*–gRNA1*_SOD1_*–gRNA2*_YPT7_* | gaattccccaacctgatgagtccgtgaggacgaaacgagtaagctcgtcgttgggaggcgcttggtgtcgttttagagctagaaatagcaagttaaaataaggctagtccgttatcaacttgaaaaagtggcaccgagtcggtgcttttggccggcatggtcccagcctcctcgctggcgccggctgggcaacatgcttcggcatggcgaatgggac**aatcactagt**aaggttctgatgagtccgtgaggacgaaacgagtaagctcgtcaaccttgatgagcaagtttggttttagagctagaaatagcaagttaaaataaggctagtccgttatcaacttgaaaaagtggcaccgagtcggtgcttttggccggcatggtcccagcctcctcgctggcgccggctgggcaacatgcttcggcatggcgaatgggac**aatcactagt**caacttctgatgagtccgtgaggacgaaacgagtaagctcgtcaagttgccggtgagcgttgagttttagagctagaaatagcaagttaaaataaggctagtccgttatcaacttgaaaaagtggcaccgagtcggtgcttttggccggcatggtcccagcctcctcgctggcgccggctgggcaacatgcttcggcatggcgaatgggacggtacc |
| T3 | gRNA1*_VPS1_*–gRNA1*_SOD1_*–gRNA5*_YPT7_* | gaattccccaacctgatgagtccgtgaggacgaaacgagtaagctcgtcgttgggaggcgcttggtgtcgttttagagctagaaatagcaagttaaaataaggctagtccgttatcaacttgaaaaagtggcaccgagtcggtgcttttggccggcatggtcccagcctcctcgctggcgccggctgggcaacatgcttcggcatggcgaatgggac**aatcactagt**aaggttctgatgagtccgtgaggacgaaacgagtaagctcgtcaaccttgatgagcaagtttggttttagagctagaaatagcaagttaaaataaggctagtccgttatcaacttgaaaaagtggcaccgagtcggtgcttttggccggcatggtcccagcctcctcgctggcgccggctgggcaacatgcttcggcatggcgaatgggac**aatcactagt**tcagaactgatgagtccgtgaggacgaaacgagtaagctcgtcttctgagtctttggagaagcgttttagagctagaaatagcaagttaaaataaggctagtccgttatcaacttgaaaaagtggcaccgagtcggtgcttttggccggcatggtcccagcctcctcgctggcgccggctgggcaacatgcttcggcatggcgaatgggacggtacc |
| T4 | gRNA1*_VPS1_*–gRNA2*_SOD1_*–gRNA1*_YPT7_* | gaattccccaacctgatgagtccgtgaggacgaaacgagtaagctcgtcgttgggaggcgcttggtgtcgttttagagctagaaatagcaagttaaaataaggctagtccgttatcaacttgaaaaagtggcaccgagtcggtgcttttggccggcatggtcccagcctcctcgctggcgccggctgggcaacatgcttcggcatggcgaatgggac**aatcactagt**aggcatctgatgagtccgtgaggacgaaacgagtaagctcgtcatgccttgcgcatgaggcatgttttagagctagaaatagcaagttaaaataaggctagtccgttatcaacttgaaaaagtggcaccgagtcggtgcttttggccggcatggtcccagcctcctcgctggcgccggctgggcaacatgcttcggcatggcgaatgggac**aatcactagt**gacattctgatgagtccgtgaggacgaaacgagtaagctcgtcaatgtcaatgtggccattccgttttagagctagaaatagcaagttaaaataaggctagtccgttatcaacttgaaaaagtggcaccgagtcggtgcttttggccggcatggtcccagcctcctcgctggcgccggctgggcaacatgcttcggcatggcgaatgggacggtacc |
| T5 | gRNA1*_VPS1_*–gRNA2*_SOD1_*–gRNA2*_YPT7_* | gaattccccaacctgatgagtccgtgaggacgaaacgagtaagctcgtcgttgggaggcgcttggtgtcgttttagagctagaaatagcaagttaaaataaggctagtccgttatcaacttgaaaaagtggcaccgagtcggtgcttttggccggcatggtcccagcctcctcgctggcgccggctgggcaacatgcttcggcatggcgaatgggac**aatcactagt**aggcatctgatgagtccgtgaggacgaaacgagtaagctcgtcatgccttgcgcatgaggcatgttttagagctagaaatagcaagttaaaataaggctagtccgttatcaacttgaaaaagtggcaccgagtcggtgcttttggccggcatggtcccagcctcctcgctggcgccggctgggcaacatgcttcggcatggcgaatgggac**aatcactagt**caacttctgatgagtccgtgaggacgaaacgagtaagctcgtcaagttgccggtgagcgttgagttttagagctagaaatagcaagttaaaataaggctagtccgttatcaacttgaaaaagtggcaccgagtcggtgcttttggccggcatggtcccagcctcctcgctggcgccggctgggcaacatgcttcggcatggcgaatgggacggtacc |
| T6 | gRNA1*_VPS1_*–gRNA2*_SOD1_*–gRNA5*_YPT7_* | gaattccccaacctgatgagtccgtgaggacgaaacgagtaagctcgtcgttgggaggcgcttggtgtcgttttagagctagaaatagcaagttaaaataaggctagtccgttatcaacttgaaaaagtggcaccgagtcggtgcttttggccggcatggtcccagcctcctcgctggcgccggctgggcaacatgcttcggcatggcgaatgggac**aatcactagt**aggcatctgatgagtccgtgaggacgaaacgagtaagctcgtcatgccttgcgcatgaggcatgttttagagctagaaatagcaagttaaaataaggctagtccgttatcaacttgaaaaagtggcaccgagtcggtgcttttggccggcatggtcccagcctcctcgctggcgccggctgggcaacatgcttcggcatggcgaatgggac**aatcactagt**tcagaactgatgagtccgtgaggacgaaacgagtaagctcgtcttctgagtctttggagaagcgttttagagctagaaatagcaagttaaaataaggctagtccgttatcaacttgaaaaagtggcaccgagtcggtgcttttggccggcatggtcccagcctcctcgctggcgccggctgggcaacatgcttcggcatggcgaatgggacggtacc |
| T7 | gRNA1*_VPS1_*–gRNA3*_SOD1_*–gRNA1*_YPT7_* | gaattccccaacctgatgagtccgtgaggacgaaacgagtaagctcgtcgttgggaggcgcttggtgtcgttttagagctagaaatagcaagttaaaataaggctagtccgttatcaacttgaaaaagtggcaccgagtcggtgcttttggccggcatggtcccagcctcctcgctggcgccggctgggcaacatgcttcggcatggcgaatgggac**aatcactagt**ggggcgctgatgagtccgtgaggacgaaacgagtaagctcgtccgccccgttcgaatgtcaacgttttagagctagaaatagcaagttaaaataaggctagtccgttatcaacttgaaaaagtggcaccgagtcggtgcttttggccggcatggtcccagcctcctcgctggcgccggctgggcaacatgcttcggcatggcgaatgggac**aatcactagt**gacattctgatgagtccgtgaggacgaaacgagtaagctcgtcaatgtcaatgtggccattccgttttagagctagaaatagcaagttaaaataaggctagtccgttatcaacttgaaaaagtggcaccgagtcggtgcttttggccggcatggtcccagcctcctcgctggcgccggctgggcaacatgcttcggcatggcgaatgggacggtacc |
| T8 | gRNA1*_VPS1_*–gRNA3*_SOD1_*–gRNA2*_YPT7_* | gaattccccaacctgatgagtccgtgaggacgaaacgagtaagctcgtcgttgggaggcgcttggtgtcgttttagagctagaaatagcaagttaaaataaggctagtccgttatcaacttgaaaaagtggcaccgagtcggtgcttttggccggcatggtcccagcctcctcgctggcgccggctgggcaacatgcttcggcatggcgaatgggac**aatcactagt**ggggcgctgatgagtccgtgaggacgaaacgagtaagctcgtccgccccgttcgaatgtcaacgttttagagctagaaatagcaagttaaaataaggctagtccgttatcaacttgaaaaagtggcaccgagtcggtgcttttggccggcatggtcccagcctcctcgctggcgccggctgggcaacatgcttcggcatggcgaatgggac**aatcactagt**caacttctgatgagtccgtgaggacgaaacgagtaagctcgtcaagttgccggtgagcgttgagttttagagctagaaatagcaagttaaaataaggctagtccgttatcaacttgaaaaagtggcaccgagtcggtgcttttggccggcatggtcccagcctcctcgctggcgccggctgggcaacatgcttcggcatggcgaatgggacggtacc |
| T9 | gRNA1*_VPS1_*–gRNA3*_SOD1_*–gRNA5*_YPT7_* | gaattccccaacctgatgagtccgtgaggacgaaacgagtaagctcgtcgttgggaggcgcttggtgtcgttttagagctagaaatagcaagttaaaataaggctagtccgttatcaacttgaaaaagtggcaccgagtcggtgcttttggccggcatggtcccagcctcctcgctggcgccggctgggcaacatgcttcggcatggcgaatgggac**aatcactagt**ggggcgctgatgagtccgtgaggacgaaacgagtaagctcgtccgccccgttcgaatgtcaacgttttagagctagaaatagcaagttaaaataaggctagtccgttatcaacttgaaaaagtggcaccgagtcggtgcttttggccggcatggtcccagcctcctcgctggcgccggctgggcaacatgcttcggcatggcgaatgggac**aatcactagt**tcagaactgatgagtccgtgaggacgaaacgagtaagctcgtcttctgagtctttggagaagcgttttagagctagaaatagcaagttaaaataaggctagtccgttatcaacttgaaaaagtggcaccgagtcggtgcttttggccggcatggtcccagcctcctcgctggcgccggctgggcaacatgcttcggcatggcgaatgggacggtacc |
| T10 | gRNA2*_VPS1_*–gRNA1*_SOD1_*–gRNA1*_YPT7_* | gaattcctttgcctgatgagtccgtgaggacgaaacgagtaagctcgtcgcaaaggtagaggtgatttcgttttagagctagaaatagcaagttaaaataaggctagtccgttatcaacttgaaaaagtggcaccgagtcggtgcttttggccggcatggtcccagcctcctcgctggcgccggctgggcaacatgcttcggcatggcgaatgggac**aatcactagt**aaggttctgatgagtccgtgaggacgaaacgagtaagctcgtcaaccttgatgagcaagtttggttttagagctagaaatagcaagttaaaataaggctagtccgttatcaacttgaaaaagtggcaccgagtcggtgcttttggccggcatggtcccagcctcctcgctggcgccggctgggcaacatgcttcggcatggcgaatgggac**aatcactagt**gacattctgatgagtccgtgaggacgaaacgagtaagctcgtcaatgtcaatgtggccattccgttttagagctagaaatagcaagttaaaataaggctagtccgttatcaacttgaaaaagtggcaccgagtcggtgcttttggccggcatggtcccagcctcctcgctggcgccggctgggcaacatgcttcggcatggcgaatgggacggtacc |
| T11 | gRNA2*_VPS1_*–gRNA1*_SOD1_*–gRNA2*_YPT7_* | gaattcctttgcctgatgagtccgtgaggacgaaacgagtaagctcgtcgcaaaggtagaggtgatttcgttttagagctagaaatagcaagttaaaataaggctagtccgttatcaacttgaaaaagtggcaccgagtcggtgcttttggccggcatggtcccagcctcctcgctggcgccggctgggcaacatgcttcggcatggcgaatgggac**aatcactagt**aaggttctgatgagtccgtgaggacgaaacgagtaagctcgtcaaccttgatgagcaagtttggttttagagctagaaatagcaagttaaaataaggctagtccgttatcaacttgaaaaagtggcaccgagtcggtgcttttggccggcatggtcccagcctcctcgctggcgccggctgggcaacatgcttcggcatggcgaatgggac**aatcactagt**caacttctgatgagtccgtgaggacgaaacgagtaagctcgtcaagttgccggtgagcgttgagttttagagctagaaatagcaagttaaaataaggctagtccgttatcaacttgaaaaagtggcaccgagtcggtgcttttggccggcatggtcccagcctcctcgctggcgccggctgggcaacatgcttcggcatggcgaatgggacggtacc |
| T12 | gRNA2*_VPS1_*–gRNA1*_SOD1_*–gRNA5*_YPT7_* | gaattcctttgcctgatgagtccgtgaggacgaaacgagtaagctcgtcgcaaaggtagaggtgatttcgttttagagctagaaatagcaagttaaaataaggctagtccgttatcaacttgaaaaagtggcaccgagtcggtgcttttggccggcatggtcccagcctcctcgctggcgccggctgggcaacatgcttcggcatggcgaatgggac**aatcactagt**aaggttctgatgagtccgtgaggacgaaacgagtaagctcgtcaaccttgatgagcaagtttggttttagagctagaaatagcaagttaaaataaggctagtccgttatcaacttgaaaaagtggcaccgagtcggtgcttttggccggcatggtcccagcctcctcgctggcgccggctgggcaacatgcttcggcatggcgaatgggac**aatcactagt**tcagaactgatgagtccgtgaggacgaaacgagtaagctcgtcttctgagtctttggagaagcgttttagagctagaaatagcaagttaaaataaggctagtccgttatcaacttgaaaaagtggcaccgagtcggtgcttttggccggcatggtcccagcctcctcgctggcgccggctgggcaacatgcttcggcatggcgaatgggacggtacc |
| T13 | gRNA2*_VPS1_*–gRNA2*_SOD1_*–gRNA1*_YPT7_* | gaattcctttgcctgatgagtccgtgaggacgaaacgagtaagctcgtcgcaaaggtagaggtgatttcgttttagagctagaaatagcaagttaaaataaggctagtccgttatcaacttgaaaaagtggcaccgagtcggtgcttttggccggcatggtcccagcctcctcgctggcgccggctgggcaacatgcttcggcatggcgaatgggac**aatcactagt**aggcatctgatgagtccgtgaggacgaaacgagtaagctcgtcatgccttgcgcatgaggcatgttttagagctagaaatagcaagttaaaataaggctagtccgttatcaacttgaaaaagtggcaccgagtcggtgcttttggccggcatggtcccagcctcctcgctggcgccggctgggcaacatgcttcggcatggcgaatgggac**aatcactagt**gacattctgatgagtccgtgaggacgaaacgagtaagctcgtcaatgtcaatgtggccattccgttttagagctagaaatagcaagttaaaataaggctagtccgttatcaacttgaaaaagtggcaccgagtcggtgcttttggccggcatggtcccagcctcctcgctggcgccggctgggcaacatgcttcggcatggcgaatgggacggtacc |
| T14 | gRNA2*_VPS1_*–gRNA2*_SOD1_*–gRNA2*_YPT7_* | gaattcctttgcctgatgagtccgtgaggacgaaacgagtaagctcgtcgcaaaggtagaggtgatttcgttttagagctagaaatagcaagttaaaataaggctagtccgttatcaacttgaaaaagtggcaccgagtcggtgcttttggccggcatggtcccagcctcctcgctggcgccggctgggcaacatgcttcggcatggcgaatgggac**aatcactagt**aggcatctgatgagtccgtgaggacgaaacgagtaagctcgtcatgccttgcgcatgaggcatgttttagagctagaaatagcaagttaaaataaggctagtccgttatcaacttgaaaaagtggcaccgagtcggtgcttttggccggcatggtcccagcctcctcgctggcgccggctgggcaacatgcttcggcatggcgaatgggac**aatcactagt**caacttctgatgagtccgtgaggacgaaacgagtaagctcgtcaagttgccggtgagcgttgagttttagagctagaaatagcaagttaaaataaggctagtccgttatcaacttgaaaaagtggcaccgagtcggtgcttttggccggcatggtcccagcctcctcgctggcgccggctgggcaacatgcttcggcatggcgaatgggacggtacc |
| T15 | gRNA2*_VPS1_*–gRNA2*_SOD1_*–gRNA5*_YPT7_* | gaattcctttgcctgatgagtccgtgaggacgaaacgagtaagctcgtcgcaaaggtagaggtgatttcgttttagagctagaaatagcaagttaaaataaggctagtccgttatcaacttgaaaaagtggcaccgagtcggtgcttttggccggcatggtcccagcctcctcgctggcgccggctgggcaacatgcttcggcatggcgaatgggac**aatcactagt**aggcatctgatgagtccgtgaggacgaaacgagtaagctcgtcatgccttgcgcatgaggcatgttttagagctagaaatagcaagttaaaataaggctagtccgttatcaacttgaaaaagtggcaccgagtcggtgcttttggccggcatggtcccagcctcctcgctggcgccggctgggcaacatgcttcggcatggcgaatgggac**aatcactagt**tcagaactgatgagtccgtgaggacgaaacgagtaagctcgtcttctgagtctttggagaagcgttttagagctagaaatagcaagttaaaataaggctagtccgttatcaacttgaaaaagtggcaccgagtcggtgcttttggccggcatggtcccagcctcctcgctggcgccggctgggcaacatgcttcggcatggcgaatgggacggtacc |
| T16 | gRNA2*_VPS1_*–gRNA3*_SOD1_*–gRNA1*_YPT7_* | gaattcctttgcctgatgagtccgtgaggacgaaacgagtaagctcgtcgcaaaggtagaggtgatttcgttttagagctagaaatagcaagttaaaataaggctagtccgttatcaacttgaaaaagtggcaccgagtcggtgcttttggccggcatggtcccagcctcctcgctggcgccggctgggcaacatgcttcggcatggcgaatgggac**aatcactagt**ggggcgctgatgagtccgtgaggacgaaacgagtaagctcgtccgccccgttcgaatgtcaacgttttagagctagaaatagcaagttaaaataaggctagtccgttatcaacttgaaaaagtggcaccgagtcggtgcttttggccggcatggtcccagcctcctcgctggcgccggctgggcaacatgcttcggcatggcgaatgggac**aatcactagt**gacattctgatgagtccgtgaggacgaaacgagtaagctcgtcaatgtcaatgtggccattccgttttagagctagaaatagcaagttaaaataaggctagtccgttatcaacttgaaaaagtggcaccgagtcggtgcttttggccggcatggtcccagcctcctcgctggcgccggctgggcaacatgcttcggcatggcgaatgggacggtacc |
| T17 | gRNA2*_VPS1_*–gRNA3*_SOD1_*–gRNA2*_YPT7_* | gaattcctttgcctgatgagtccgtgaggacgaaacgagtaagctcgtcgcaaaggtagaggtgatttcgttttagagctagaaatagcaagttaaaataaggctagtccgttatcaacttgaaaaagtggcaccgagtcggtgcttttggccggcatggtcccagcctcctcgctggcgccggctgggcaacatgcttcggcatggcgaatgggac**aatcactagt**ggggcgctgatgagtccgtgaggacgaaacgagtaagctcgtccgccccgttcgaatgtcaacgttttagagctagaaatagcaagttaaaataaggctagtccgttatcaacttgaaaaagtggcaccgagtcggtgcttttggccggcatggtcccagcctcctcgctggcgccggctgggcaacatgcttcggcatggcgaatgggac**aatcactagt**caacttctgatgagtccgtgaggacgaaacgagtaagctcgtcaagttgccggtgagcgttgagttttagagctagaaatagcaagttaaaataaggctagtccgttatcaacttgaaaaagtggcaccgagtcggtgcttttggccggcatggtcccagcctcctcgctggcgccggctgggcaacatgcttcggcatggcgaatgggacggtacc |
| T18 | gRNA2*_VPS1_*–gRNA3*_SOD1_*–gRNA5*_YPT7_* | gaattcctttgcctgatgagtccgtgaggacgaaacgagtaagctcgtcgcaaaggtagaggtgatttcgttttagagctagaaatagcaagttaaaataaggctagtccgttatcaacttgaaaaagtggcaccgagtcggtgcttttggccggcatggtcccagcctcctcgctggcgccggctgggcaacatgcttcggcatggcgaatgggac**aatcactagt**ggggcgctgatgagtccgtgaggacgaaacgagtaagctcgtccgccccgttcgaatgtcaacgttttagagctagaaatagcaagttaaaataaggctagtccgttatcaacttgaaaaagtggcaccgagtcggtgcttttggccggcatggtcccagcctcctcgctggcgccggctgggcaacatgcttcggcatggcgaatgggac**aatcactagt**tcagaactgatgagtccgtgaggacgaaacgagtaagctcgtcttctgagtctttggagaagcgttttagagctagaaatagcaagttaaaataaggctagtccgttatcaacttgaaaaagtggcaccgagtcggtgcttttggccggcatggtcccagcctcctcgctggcgccggctgggcaacatgcttcggcatggcgaatgggacggtacc |
| T19 | gRNA3*_VPS1_*–gRNA1*_SOD1_*–gRNA1*_YPT7_* | gaattcctctcgctgatgagtccgtgaggacgaaacgagtaagctcgtccgagagggtgaccagtagacgttttagagctagaaatagcaagttaaaataaggctagtccgttatcaacttgaaaaagtggcaccgagtcggtgcttttggccggcatggtcccagcctcctcgctggcgccggctgggcaacatgcttcggcatggcgaatgggac**aatcactagt**aaggttctgatgagtccgtgaggacgaaacgagtaagctcgtcaaccttgatgagcaagtttggttttagagctagaaatagcaagttaaaataaggctagtccgttatcaacttgaaaaagtggcaccgagtcggtgcttttggccggcatggtcccagcctcctcgctggcgccggctgggcaacatgcttcggcatggcgaatgggac**aatcactagt**gacattctgatgagtccgtgaggacgaaacgagtaagctcgtcaatgtcaatgtggccattccgttttagagctagaaatagcaagttaaaataaggctagtccgttatcaacttgaaaaagtggcaccgagtcggtgcttttggccggcatggtcccagcctcctcgctggcgccggctgggcaacatgcttcggcatggcgaatgggacggtacc |
| T20 | gRNA3*_VPS1_*–gRNA1*_SOD1_*–gRNA2*_YPT7_* | gaattcctctcgctgatgagtccgtgaggacgaaacgagtaagctcgtccgagagggtgaccagtagacgttttagagctagaaatagcaagttaaaataaggctagtccgttatcaacttgaaaaagtggcaccgagtcggtgcttttggccggcatggtcccagcctcctcgctggcgccggctgggcaacatgcttcggcatggcgaatgggac**aatcactagt**aaggttctgatgagtccgtgaggacgaaacgagtaagctcgtcaaccttgatgagcaagtttggttttagagctagaaatagcaagttaaaataaggctagtccgttatcaacttgaaaaagtggcaccgagtcggtgcttttggccggcatggtcccagcctcctcgctggcgccggctgggcaacatgcttcggcatggcgaatgggac**aatcactagt**caacttctgatgagtccgtgaggacgaaacgagtaagctcgtcaagttgccggtgagcgttgagttttagagctagaaatagcaagttaaaataaggctagtccgttatcaacttgaaaaagtggcaccgagtcggtgcttttggccggcatggtcccagcctcctcgctggcgccggctgggcaacatgcttcggcatggcgaatgggacggtacc |
| T21 | gRNA3*_VPS1_*–gRNA1*_SOD1_*–gRNA5*_YPT7_* | gaattcctctcgctgatgagtccgtgaggacgaaacgagtaagctcgtccgagagggtgaccagtagacgttttagagctagaaatagcaagttaaaataaggctagtccgttatcaacttgaaaaagtggcaccgagtcggtgcttttggccggcatggtcccagcctcctcgctggcgccggctgggcaacatgcttcggcatggcgaatgggac**aatcactagt**aaggttctgatgagtccgtgaggacgaaacgagtaagctcgtcaaccttgatgagcaagtttggttttagagctagaaatagcaagttaaaataaggctagtccgttatcaacttgaaaaagtggcaccgagtcggtgcttttggccggcatggtcccagcctcctcgctggcgccggctgggcaacatgcttcggcatggcgaatgggac**aatcactagt**tcagaactgatgagtccgtgaggacgaaacgagtaagctcgtcttctgagtctttggagaagcgttttagagctagaaatagcaagttaaaataaggctagtccgttatcaacttgaaaaagtggcaccgagtcggtgcttttggccggcatggtcccagcctcctcgctggcgccggctgggcaacatgcttcggcatggcgaatgggacggtacc |
| T22 | gRNA3*_VPS1_*–gRNA2*_SOD1_*–gRNA1*_YPT7_* | gaattcctctcgctgatgagtccgtgaggacgaaacgagtaagctcgtccgagagggtgaccagtagacgttttagagctagaaatagcaagttaaaataaggctagtccgttatcaacttgaaaaagtggcaccgagtcggtgcttttggccggcatggtcccagcctcctcgctggcgccggctgggcaacatgcttcggcatggcgaatgggac**aatcactagt**aggcatctgatgagtccgtgaggacgaaacgagtaagctcgtcatgccttgcgcatgaggcatgttttagagctagaaatagcaagttaaaataaggctagtccgttatcaacttgaaaaagtggcaccgagtcggtgcttttggccggcatggtcccagcctcctcgctggcgccggctgggcaacatgcttcggcatggcgaatgggac**aatcactagt**gacattctgatgagtccgtgaggacgaaacgagtaagctcgtcaatgtcaatgtggccattccgttttagagctagaaatagcaagttaaaataaggctagtccgttatcaacttgaaaaagtggcaccgagtcggtgcttttggccggcatggtcccagcctcctcgctggcgccggctgggcaacatgcttcggcatggcgaatgggacggtacc |
| T23 | gRNA3*_VPS1_*–gRNA2*_SOD1_*–gRNA2*_YPT7_* | gaattcctctcgctgatgagtccgtgaggacgaaacgagtaagctcgtccgagagggtgaccagtagacgttttagagctagaaatagcaagttaaaataaggctagtccgttatcaacttgaaaaagtggcaccgagtcggtgcttttggccggcatggtcccagcctcctcgctggcgccggctgggcaacatgcttcggcatggcgaatgggac**aatcactagt**aggcatctgatgagtccgtgaggacgaaacgagtaagctcgtcatgccttgcgcatgaggcatgttttagagctagaaatagcaagttaaaataaggctagtccgttatcaacttgaaaaagtggcaccgagtcggtgcttttggccggcatggtcccagcctcctcgctggcgccggctgggcaacatgcttcggcatggcgaatgggac**aatcactagt**caacttctgatgagtccgtgaggacgaaacgagtaagctcgtcaagttgccggtgagcgttgagttttagagctagaaatagcaagttaaaataaggctagtccgttatcaacttgaaaaagtggcaccgagtcggtgcttttggccggcatggtcccagcctcctcgctggcgccggctgggcaacatgcttcggcatggcgaatgggacggtacc |
| T24 | gRNA3*_VPS1_*–gRNA2*_SOD1_*–gRNA5*_YPT7_* | gaattcctctcgctgatgagtccgtgaggacgaaacgagtaagctcgtccgagagggtgaccagtagacgttttagagctagaaatagcaagttaaaataaggctagtccgttatcaacttgaaaaagtggcaccgagtcggtgcttttggccggcatggtcccagcctcctcgctggcgccggctgggcaacatgcttcggcatggcgaatgggac**aatcactagt**aggcatctgatgagtccgtgaggacgaaacgagtaagctcgtcatgccttgcgcatgaggcatgttttagagctagaaatagcaagttaaaataaggctagtccgttatcaacttgaaaaagtggcaccgagtcggtgcttttggccggcatggtcccagcctcctcgctggcgccggctgggcaacatgcttcggcatggcgaatgggac**aatcactagt**tcagaactgatgagtccgtgaggacgaaacgagtaagctcgtcttctgagtctttggagaagcgttttagagctagaaatagcaagttaaaataaggctagtccgttatcaacttgaaaaagtggcaccgagtcggtgcttttggccggcatggtcccagcctcctcgctggcgccggctgggcaacatgcttcggcatggcgaatgggacggtacc |
| T25 | gRNA3*_VPS1_*–gRNA3*_SOD1_*–gRNA1*_YPT7_* | gaattcctctcgctgatgagtccgtgaggacgaaacgagtaagctcgtccgagagggtgaccagtagacgttttagagctagaaatagcaagttaaaataaggctagtccgttatcaacttgaaaaagtggcaccgagtcggtgcttttggccggcatggtcccagcctcctcgctggcgccggctgggcaacatgcttcggcatggcgaatgggac**aatcactagt**ggggcgctgatgagtccgtgaggacgaaacgagtaagctcgtccgccccgttcgaatgtcaacgttttagagctagaaatagcaagttaaaataaggctagtccgttatcaacttgaaaaagtggcaccgagtcggtgcttttggccggcatggtcccagcctcctcgctggcgccggctgggcaacatgcttcggcatggcgaatgggac**aatcactagt**gacattctgatgagtccgtgaggacgaaacgagtaagctcgtcaatgtcaatgtggccattccgttttagagctagaaatagcaagttaaaataaggctagtccgttatcaacttgaaaaagtggcaccgagtcggtgcttttggccggcatggtcccagcctcctcgctggcgccggctgggcaacatgcttcggcatggcgaatgggacggtacc |
| T26 | gRNA3*_VPS1_*–gRNA3*_SOD1_*–gRNA2*_YPT7_* | gaattcctctcgctgatgagtccgtgaggacgaaacgagtaagctcgtccgagagggtgaccagtagacgttttagagctagaaatagcaagttaaaataaggctagtccgttatcaacttgaaaaagtggcaccgagtcggtgcttttggccggcatggtcccagcctcctcgctggcgccggctgggcaacatgcttcggcatggcgaatgggac**aatcactagt**ggggcgctgatgagtccgtgaggacgaaacgagtaagctcgtccgccccgttcgaatgtcaacgttttagagctagaaatagcaagttaaaataaggctagtccgttatcaacttgaaaaagtggcaccgagtcggtgcttttggccggcatggtcccagcctcctcgctggcgccggctgggcaacatgcttcggcatggcgaatgggac**aatcactagt**caacttctgatgagtccgtgaggacgaaacgagtaagctcgtcaagttgccggtgagcgttgagttttagagctagaaatagcaagttaaaataaggctagtccgttatcaacttgaaaaagtggcaccgagtcggtgcttttggccggcatggtcccagcctcctcgctggcgccggctgggcaacatgcttcggcatggcgaatgggacggtacc |
| T27 | gRNA3*_VPS1_*–gRNA3*_SOD1_*–gRNA5*_YPT7_* | gaattcctctcgctgatgagtccgtgaggacgaaacgagtaagctcgtccgagagggtgaccagtagacgttttagagctagaaatagcaagttaaaataaggctagtccgttatcaacttgaaaaagtggcaccgagtcggtgcttttggccggcatggtcccagcctcctcgctggcgccggctgggcaacatgcttcggcatggcgaatgggac**aatcactagt**ggggcgctgatgagtccgtgaggacgaaacgagtaagctcgtccgccccgttcgaatgtcaacgttttagagctagaaatagcaagttaaaataaggctagtccgttatcaacttgaaaaagtggcaccgagtcggtgcttttggccggcatggtcccagcctcctcgctggcgccggctgggcaacatgcttcggcatggcgaatgggac**aatcactagt**tcagaactgatgagtccgtgaggacgaaacgagtaagctcgtcttctgagtctttggagaagcgttttagagctagaaatagcaagttaaaataaggctagtccgttatcaacttgaaaaagtggcaccgagtcggtgcttttggccggcatggtcccagcctcctcgctggcgccggctgggcaacatgcttcggcatggcgaatgggacggtacc |
