# Supplementary material for: Multiplexed CRISPR-mediated engineering of protein secretory pathway genes in the thermotolerant methylotrophic yeast Ogataea thermomethanolica
Source: PLoS One. 2021 Dec 23;16(12):e0261754. doi: 10.1371/journal.pone.0261754 (PMC8699913; doi:10.1371/journal.pone.0261754)
Supplement: S3 Table — (DOCX) [file pone.0261754.s007.docx]

**Table S3** **List of primer sequences used in this study.**

| **Genes** | **Names** | **Sequences (5′-3′)** | **Ta (°C)** | **Sources** |
| --- | --- | --- | --- | --- |
| **Hygromycin resistance gene** | | |  |  |
| *Hyg*^R^ | Hyg-F | ATGAAAAAGCCTGAACTCACC | 55.0 | [23] |
|  | Hyg-R | TCCATCACAGTTTGCCAGTG |  |  |
| **pOtAOX-gRNA integration** | | |  |  |
|  | OtAOX-F | CCAATGCATGCACAAGCTGGACGAGTCGC | 55.0 | [23] |
|  | OtAOX-R | GCAAATGGCATTCTGACATCC |  |  |
| **Target protein secretory pathway genes** | | |  |  |
| *SOD1* | Sod1-F | GGAGACTCCACTGTCAAGGG | 56.3 | This study |
|  | Sod1-R | TCAAGCAGGAAAGCCAATG |  |  |
| *VPS1* | Vps1-F | ATGGATGAAACATTAATCCAAACC | 56.3 | This study |
|  | Vps1-R | TTGGTCAGTCCAGGCAAATC |  |  |
| *YPT7* | Ypt7-F | GCAAATATGGGACACTGCTG | 56.3 | This study |
|  | Ypt7-R | CTGAACTCGAGACTCTCCTC |  |  |
| *YPT35* | Ypt35-F | ATGTCTGAACACACACATTTGAA | 56.3 | This study |
|  | Ypt35-R | CTAATTCTCGCTAATTAGCTTCTGG |  |  |
| **RT-qPCR** | | |  |  |
| *ACT* | RT-Act-F | CTTTCAACGTTCCAGCTTTC | 58.5 | [23] |
|  | RT-Act-R | AGGAACAACGTGGGTAACAC |  |  |
| *SOD1* | RT-Sod1-F | CCACTGTCAAGGGAATTGTT | 58.5 | [23] |
|  | RT-Sod1-R | AATTGATGGATGTGGAAACC |  |  |
| *VPS1* | RT-Vps1-F | CCAATGAGTTGAGTGGAGGT | 58.5 | [23] |
|  | RT-Vps1-R | GTCTGCATCCTTGATCTGGT |  |  |
| *YPT7* | RT-Ypt7-F | TTGTGTACGATGTCACCAAC | 55.0 | [23] |
|  | RT-Ypt7-R | CAAACGGGAAGTTATCAGGA |  |  |
| *YPT35* | RT-Ypt35-F | CCGAGCCAACTCTCATCAAA | 53.0 | This study |
|  | RT-Ypt35-R | GGAAGTTCTGTTTGGACGGT |  |  |

**Remark:** Ta, annealing temperature

**Reference**

23. Kruasuwan W, Puseenam A, Phithakrotchanakoon C, Tanapongpipat S, and Roongsawang N. Modulation of heterologous protein secretion in the thermotolerant methylotrophic yeast *Ogataea thermomethanolica* TBRC 656 by CRISPR-Cas9 system. PLoS ONE. 2021;16:e0258005. doi: https://doi.org/10.1371/journal.pone. 0258005.
