# Supplementary material for: Multiplexed CRISPR-mediated engineering of protein secretory pathway genes in the thermotolerant methylotrophic yeast Ogataea thermomethanolica
Source: PLoS One. 2021 Dec 23;16(12):e0261754. doi: 10.1371/journal.pone.0261754 (PMC8699913; doi:10.1371/journal.pone.0261754)
Supplement: S4 Table — O. thermomethanolica ACT was used to normalize gene expression. Data are shown as mean ± S.D. from three independent biological replicate experiments (n = 3). (DOCX) [file pone.0261754.s008.docx]

**Table S4 Relative gene expression levels of simultaneous gene disrupted mutants.**

*O*. *thermomethanolica* *ACT* was used to normalize gene expression. Data are shown as mean ± S.D. from three independent biological replicate experiments (*n*=3).

| **Name (genes)** | **Clones no.** | **Genes** | **Relative expression (FC)** |
| --- | --- | --- | --- |
| Ot-Cas9-Xyl | Control |  | 1.0 ± 0.15 |
| 2G  (*vps1*–*sod1*) | 2 | *VPS1* | 0.8 ± 0.07 |
|  |  | *SOD1* | 0.2 ± 0.01 |
|  | 4 | *VPS1* | 0.7 ± 0.14 |
|  |  | *SOD1* | 0.2 ± 0.01 |
| 3G  (*vps1*–*sod1*–*ypt35*) | 3 | *VPS1* | 0.7 ± 0.39 |
|  |  | *SOD1* | 0.5 ± 0.03 |
|  |  | *YPT35* | 0.1 ± 0.05 |
|  | 4 | *VPS1* | 0.3 ± 0.03 |
|  |  | *SOD1* | 0.2 ± 0.01 |
|  |  | *YPT35* | 0.1 ± 0.13 |
|  | 5 | *VPS1* | 0.3 ± 0.04 |
|  |  | *SOD1* | 0.3 ± 0.10 |
|  |  | *YPT35* | 0.3 ± 0.05 |
| 4G  (*vps1*–*sod1*–*ypt35*–*ypt7*) | 2 | *VPS1* | 0.3 ± 0.01 |
|  |  | *SOD1* | 0.8 ± 0.03 |
|  |  | *YPT35* | 0.3 ± 0.14 |
|  |  | *YPT7* | 0.1 ± 0.02 |
