# Supplementary material for: Multiplexed CRISPR-mediated engineering of protein secretory pathway genes in the thermotolerant methylotrophic yeast Ogataea thermomethanolica
Source: PLoS One. 2021 Dec 23;16(12):e0261754. doi: 10.1371/journal.pone.0261754 (PMC8699913; doi:10.1371/journal.pone.0261754)
Supplement: S5 Table — Data are shown as mean ± S.D. from three independent biological replicate experiments (n = 3). (DOCX) [file pone.0261754.s009.docx]

**Table S5 Xylanase activity of Ot-dCas9-VP64-Xyl expressing various gRNAs (T1-T27).**

Data are shown as mean ± S.D. from three independent biological replicate experiments (*n*=3).

| **Strains (gRNAs)** | **No.** | **Relative activity (%U/OD)** | |
| --- | --- | --- | --- |
| Ot-dCas9-VP64-Xyl (control) |  | 100.0 | 100 ± 1.79 |
| T1 | 1 | 118.8 | 117 ± 7.42 |
| (gRNA1*_VPS1_*–gRNA1*_SOD1_*–gRNA1*_YPT7_*) | 2 | 107.3 |  |
|  | 3 | 123.4 |  |
| T2 | 1 | 68.7 | 83 ± 3.68 |
| (gRNA1*_VPS1_*–gRNA1*_SOD1_*–gRNA2*_YPT7_*) | 2 | 94.3 |  |
|  | 3 | 86.8 |  |
| T3 | 1 | 101.4 | 105 ± 5.79 |
| (gRNA1*_VPS1_*–gRNA1*_SOD1_*–gRNA5*_YPT7_*) | 2 | 107.5 |  |
|  | 3 | 107.2 |  |
| T4 | 1 | 95.4 | 98 ± 2.17 |
| (gRNA1*_VPS1_*–gRNA2*_SOD1_*–gRNA1*_YPT7_*) | 2 | 93.0 |  |
|  | 3 | 105.9 |  |
| T5 | 1 | 92.6 | 92 ± 2.63 |
| (gRNA1*_VPS1_*–gRNA2*_SOD1_*–gRNA2*_YPT7_*) | 2 | 87.2 |  |
|  | 3 | 96.6 |  |
| T6 | 1 | 140.4 | 129 ± 11.67 |
| (gRNA1*_VPS1_*–gRNA2*_SOD1_*–gRNA5*_YPT7_*) | 2 | 125.6 |  |
|  | 3 | 119.8 |  |
| T7 | 1 | 100.4 | 101 ± 3.42 |
| (gRNA1*_VPS1_*–gRNA3*_SOD1_*–gRNA1*_YPT7_*) | 2 | 101.4 |  |
|  | 3 | 102.1 |  |
| T8 | 1 | 99.5 | 105 ± 1.01 |
| (gRNA1*_VPS1_*–gRNA3*_SOD1_*–gRNA2*_YPT7_*) | 2 | 108.8 |  |
|  | 3 | 106.0 |  |
| T9 | 1 | 100.1 | 98 ± 1.81 |
| (gRNA1*_VPS1_*–gRNA3*_SOD1_*–gRNA5*_YPT7_*) | 2 | 92.1 |  |
|  | 3 | 101.8 |  |
| T10 | 1 | 149.4 | 129 ± 9.52 |
| (gRNA2*_VPS1_*–gRNA1*_SOD1_*–gRNA1*_YPT7_*) | 2 | 116.7 |  |
|  | 3 | 120.5 |  |
| T11 | 1 | 93.78 | 95 ± 3.49 |
| (gRNA2*_VPS1_*–gRNA1*_SOD1_*–gRNA2*_YPT7_*) | 2 | 101.33 |  |
|  | 3 | 89.50 |  |
| T12 | 1 | 99.0 | 99 ± 3.09 |
| (gRNA2*_VPS1_*–gRNA1*_SOD1_*–gRNA5*_YPT7_*) | 2 | 95.8 |  |
|  | 3 | 102.0 |  |
| T13 | 1 | 97.6 | 112 ± 11.39 |
| (gRNA2*_VPS1_*–gRNA2*_SOD1_*–gRNA1*_YPT7_*) | 2 | 121.4 |  |
|  | 3 | 118.3 |  |

| T14 | 1 | 97.7 | 106 ± 6.40 |
| --- | --- | --- | --- |
| (gRNA2*_VPS1_*–gRNA2*_SOD1_*–gRNA2*_YPT7_*) | 2 | 107.7 |  |
|  | 3 | 113.1 |  |
| T15 | 1 | 107.4 | 109 ± 7.04 |
| (gRNA2*_VPS1_*–gRNA2*_SOD1_*–gRNA5*_YPT7_*) | 2 | 104.3 |  |
|  | 3 | 114.8 |  |
| T16 | 1 | 108.9 | 109 ± 6.81 |
| (gRNA2*_VPS1_*–gRNA3*_SOD1_*–gRNA1*_YPT7_*) | 2 | 106.7 |  |
|  | 3 | 110.7 |  |
| T17 | 1 | 84.3 | 87 ± 8.01 |
| (gRNA2*_VPS1_*–gRNA3*_SOD1_*–gRNA2*_YPT7_*) | 2 | 76.3 |  |
|  | 3 | 99.2 |  |
| T18 | 1 | 134.6 | 121 ± 10.22 |
| (gRNA2*_VPS1_*–gRNA3*_SOD1_*–gRNA5*_YPT7_*) | 2 | 113.6 |  |
|  | 3 | 114.2 |  |
| T19 | 1 | 116.6 | 114 ± 4.87 |
| (gRNA3*_VPS1_*–gRNA1*_SOD1_*–gRNA1*_YPT7_*) | 2 | 113.6 |  |
|  | 3 | 113.0 |  |
| T20 | 1 | 98.8 | 97 ± 4.83 |
| (gRNA3*_VPS1_*–gRNA1*_SOD1_*–gRNA2*_YPT7_*) | 2 | 99.7 |  |
|  | 3 | 93.9 |  |
| T21 | 1 | 104.4 | 107 ± 4.21 |
| (gRNA3*_VPS1_*–gRNA1*_SOD1_*–gRNA5*_YPT7_*) | 2 | 107.1 |  |
|  | 3 | 109.9 |  |
| T22 | 1 | 115.7 | 112 ± 5.13 |
| (gRNA3*_VPS1_*–gRNA2*_SOD1_*–gRNA1*_YPT7_*) | 2 | 107.2 |  |
|  | 3 | 112.3 |  |
| T23 | 1 | 113.7 | 118 ± 6.59 |
| (gRNA3*_VPS1_*–gRNA2*_SOD1_*–gRNA2*_YPT7_*) | 2 | 120.0 |  |
|  | 3 | 120.6 |  |
| T24 | 1 | 113.2 | 114 ± 6.47 |
| (gRNA3*_VPS1_*–gRNA2*_SOD1_*–gRNA5*_YPT7_*) | 2 | 111.6 |  |
|  | 3 | 116.5 |  |
| T25 | 1 | 120.0 | 118 ± 2.14 |
| (gRNA3*_VPS1_*–gRNA3*_SOD1_*–gRNA1*_YPT7_*) | 2 | 116.3 |  |
|  | 3 | 117.7 |  |
| T26 | 1 | 91.6 | 95 ± 1.13 |
| (gRNA3*_VPS1_*–gRNA3*_SOD1_*–gRNA2*_YPT7_*) | 2 | 94.8 |  |
|  | 3 | 98.7 |  |
| T27 | 1 | 111.0 | 115 ± 4.75 |
| (gRNA3*_VPS1_*–gRNA3*_SOD1_*–gRNA5*_YPT7_*) | 2 | 125.0 |  |
|  | 3 | 109.3 |  |
