# Supplementary material for: Multiplexed CRISPR-mediated engineering of protein secretory pathway genes in the thermotolerant methylotrophic yeast Ogataea thermomethanolica
Source: PLoS One. 2021 Dec 23;16(12):e0261754. doi: 10.1371/journal.pone.0261754 (PMC8699913; doi:10.1371/journal.pone.0261754)
Supplement: S7 Table — Data are shown as mean ± S.D. from three-independent biological replicate experiments (n = 3). (DOCX) [file pone.0261754.s011.docx]

**Table S7 Phytase activity of Ot-dCas9-VP64-Phy expressing T6, T10 and T18 gRNAs.** Data are shown as mean ± S.D. from three-independent biological replicate experiments (*n*=3).

| **Strains (gRNAs)** | **No.** | **Relative activity (%U/OD)** | |
| --- | --- | --- | --- |
| Ot-dCas9-VP64-Phy (control) |  |  | 100 ± 0.92 |
| T6 | 1 | 72.3 ± 2.28 | 90 ± 30.45 |
| (gRNA1*_VPS1_*–gRNA2*_SOD1_*–gRNA5*_YPT7_*) | 2 | 72.8 ± 1.66 |  |
|  | 3 | 125.3 ± 4.63 |  |
| T10 | 1 | 134.7 ± 9.39 | 141 ± 22.71 |
| (gRNA2*_VPS1_*–gRNA1*_SOD1_*–gRNA1*_YPT7_*) | 2 | 122.5 ± 7.76 |  |
|  | 3 | 166.5 ± 6.47 |  |
| T18 | 1 | 73.0 ± 2.17 | 72 ± 1.13 |
| (gRNA2*_VPS1_*­–gRNA3*_SOD1_*–gRNA5*_YPT7_*) | 2 | 71.1 ± 6.51 |  |
|  | 3 | 73.2 ± 1.58 |  |
