# Supplementary material for: Multiplexed CRISPR-mediated engineering of protein secretory pathway genes in the thermotolerant methylotrophic yeast Ogataea thermomethanolica
Source: PLoS One. 2021 Dec 23;16(12):e0261754. doi: 10.1371/journal.pone.0261754 (PMC8699913; doi:10.1371/journal.pone.0261754)
Supplement: S8 Table — O. thermomethanolica ACT was used to normalize gene expression. Data are shown as mean ± S.D. from three independent biological replicate experiments (n = 3). (DOCX) [file pone.0261754.s012.docx]

**Table S8 Relative gene expression levels of Ot-dCas9-VP64-Phy expressing T6, T10 and T18 gRNAs.** *O*. *thermomethanolica* *ACT* was used to normalize gene expression. Data are shown as mean ± S.D. from three independent biological replicate experiments (*n*=3).

| **Strains (gRNAs)** | **Genes** | **Relative expression (FC)** |
| --- | --- | --- |
| Ot-dCas9-VP64-Phy (control) |  | 1.0 ± 0.10 |
| T6 | *VPS1* | 1.1 ± 0.32 |
| (gRNA1*_VPS1_*–gRNA2*_SOD1_*–gRNA5*_YPT7_*) | *SOD1* | 0.9 ± 0.13 |
|  | *YPT7* | 1.5 ± 0.66 |
| T10 | *VPS1* | 1.8 ± 0.14 |
| (gRNA2*_VPS1_*–gRNA1*_SOD1_*–gRNA1*_YPT7_*) | *SOD1* | 2.5 ± 0.21 |
|  | *YPT7* | 1.2 ± 0.14 |
| T18 | *VPS1* | 1.2 ± 0.34 |
| (gRNA2*_VPS1_*–gRNA3*_SOD1_*–gRNA5*_YPT7_*) | *SOD1* | 1.8 ± 0.30 |
|  | *YPT7* | 1.4 ± 0.08 |
